# Smart Agriculture Research in Date Palm (*Phoenix dactylifera* L.) Production: A Comprehensive Bibliometric Study

**DOI:** 10.3390/plants15203137

**Published:** 2026-10-14

**Authors:** Sajid Safeer, Adnan Nadeem, Sajid Maqsood, Cataldo Pulvento

**Affiliations:** 1 Department of Soil, Food and Plant Sciences, University of Bari Aldo Moro, 70126 Bari, Italy; 2 Faculty of Computer and Information System, Islamic University of Madinah, 42351 Medina, Saudi Arabia; 3 Department of Food Science, College of Agriculture and Veterinary Medicine, United Arab Emirates University, 15551 Al Ain, United Arab Emirates; sajid.m@uaeu.ac.ae

**Keywords:** bibliometric, smart agriculture, thematic analysis, Arabian Peninsula, *Phoenix dactylifera*

## Abstract

The date palm (*Phoenix dactylifera* L.) is fundamental to food security, livelihoods, and agro-ecosystem sustainability across the Arabian Peninsula, yet its production is increasingly constrained by water scarcity, salinity, climate change, and biotic stresses. Smart agriculture technologies offer opportunities to improve crop monitoring, resource-use efficiency, and decision-making; however, their application in date palm production remains fragmented and insufficiently validated. This study evaluates the development, geographic distribution, technological focus, research approaches, and thematic structure of smart agriculture research on date palms in the Arabian Peninsula and other major date-producing countries. A bibliometric analysis was conducted using peer-reviewed English-language articles and reviews indexed in Scopus and Web of Science from 2016 to 2026, with 2026 representing a partial year. Following screening and duplicate removal, 164 published studies were retained. Publication output increased gradually, although the trend was not statistically significant (β = 1.25 articles year^−1^, *p* = 0.068; Mann–Kendall τ = 0.489, *p* = 0.060). The literature was concentrated in the Gulf Cooperation Council (GCC) region, with Saudi Arabia contributing 54 publications (32.9%). General agronomy/other topics accounted for 46.3% of publications, while remote sensing/UAVs (17.1%), GIS/geospatial technologies (14.0%), IoT/sensors (13.4%), and AI/machine learning (9.8%) represented the leading specific technology categories. Water and irrigation management dominated thematic applications (46.3%), whereas Smart Farming/IoT accounted for only 11.0%. Observational and monitoring studies comprised 50.0% of publications, compared with only 6.1% involving field experiments, indicating limited field-scale validation. Internationally co-authored studies received significantly more citations than domestically authored studies (16.55 vs. 8.68; *p* = 0.0007). Thematic analysis identified four major clusters: geospatial monitoring, water management, pest control, and sustainability, with AI/machine learning showing marked growth after 2022. Altogether, the field is expanding but remains fragmented, highlighting the need for integrated IoT and sensor networks, rigorous field trials, AI-driven decision-support systems, and stronger regional and international collaboration to enable scalable, context-specific smart agriculture for date palm production.

## 1. Introduction

### 1.1. Date Palm Production in the Arabian Peninsula

The Arabian Peninsula represents one of the world’s most challenging environments for agricultural production. The region is characterized by arid and hyperarid climates, low and highly variable rainfall, high temperatures, limited arable land, and widespread soil and groundwater salinity [1,2,3,4]. Annual precipitation across much of the Peninsula is below 100 mm, while temperatures in inland areas can exceed 45–50 °C during summer [3]. These environmental constraints, together with increasing water scarcity, groundwater depletion and the projected effects of climate change, place substantial pressure on agricultural productivity and resource-use efficiency [5,6,7].

Within this context, the date palm (*Phoenix dactylifera* L.) is a particularly important crop. Its long history of cultivation and adaptation to high temperatures, water-limited environments and saline conditions have enabled date palm production to become an integral component of agricultural systems across the Arabian Peninsula and neighboring arid regions [8,9,10]. The crop contributes to food supply, agricultural livelihoods and rural economies, while its fruit and numerous biomass-derived products provide additional nutritional and economic value [11,12,13]. Saudi Arabia, the United Arab Emirates (UAE), Oman and other countries in the region are among the world’s major date-producing areas, making the sustainability and productivity of date palm cultivation strategically important for regional agriculture and food security [14,15,16].

Despite its adaptation to arid environments, date palm production is increasingly exposed to interacting biotic, abiotic and socioeconomic constraints. Water scarcity and salinity directly affect plant growth and productivity, while increasing temperatures and climatic variability may further intensify these stresses [17,18,19]. Pests and diseases, particularly the red palm weevil (*Rhynchophorus ferrugineus*), represent major production threats requiring continuous monitoring and timely intervention [20,21]. In addition, labor-intensive operations, postharvest losses and variability in farm management contribute to inefficiencies across the production chain [22,23,24]. These challenges create a strong need for more precise, timely and resource-efficient approaches to crop monitoring and management.

### 1.2. Smart Agriculture and Date Palm Production

Smart agriculture provides a technological framework for addressing several of these challenges through the integration of sensing, communication, geospatial, computational and decision-support technologies. In agricultural systems, Internet of Things (IoT) sensors can monitor soil moisture, salinity and microclimatic conditions; remote sensing and unmanned aerial vehicles (UAVs) can support crop mapping and stress assessment; geographic information systems (GIS) can facilitate spatial analysis and resource planning; and artificial intelligence (AI) and machine learning (ML) can enable automated analysis, prediction and detection of pests, diseases and crop conditions [25,26,27,28].

These technologies have particular relevance to date palm production because many management decisions are strongly influenced by spatial and temporal variability. For example, irrigation requirements vary according to soil properties, environmental conditions, cultivar and palm age, while early detection of pests and diseases is important for reducing plantation-level losses. Data-driven technologies can therefore complement conventional management by enabling more continuous monitoring, site-specific interventions and evidence-based decision-making.

However, the adoption of smart agriculture technologies in date palm systems remains uneven. Technologies developed and validated in other agricultural systems cannot necessarily be transferred directly to the Arabian Peninsula, where extreme heat, dust, salinity, water scarcity, heterogeneous plantation structures and variable connectivity can affect technological performance and deployment [29,30,31]. Moreover, the implementation of digital agriculture requires not only technological capacity but also appropriate infrastructure, technical expertise, economic feasibility and integration with existing farming practices. Understanding how these technologies are currently being investigated and applied in date palm systems is therefore important for identifying both progress and persistent limitations.

### 1.3. Research Landscape and Knowledge Gap

Research on date palm agriculture has expanded across multiple disciplines, including water and irrigation management, soil and salinity assessment, remote sensing, pest and disease management, genetics and breeding, fruit quality and agricultural sustainability. More recently, technologies such as IoT, UAVs, GIS, AI and ML have begun to appear within this research landscape. Nevertheless, the literature is dispersed across agricultural, environmental, engineering and technological journals and encompasses diverse methodological approaches and research objectives.

Therefore, the overall structure and evolution of research on smart agriculture for date palm production in the Arabian Peninsula remain insufficiently characterized. In particular, there is limited quantitative evidence concerning how research output has evolved over time, which countries and institutions contribute most strongly, how international collaboration is structured, which technologies and research topics receive the greatest attention, and where methodological or technological gaps persist. The distinction between established research domains, emerging technologies and underexplored areas is also difficult to assess from individual studies alone.

Bibliometric analysis provides a systematic approach for addressing these issues by quantitatively mapping publication trends, geographic contributions, collaboration patterns, citation structures, research topics and technological domains within a defined body of literature [32,33,34]. When combined with thematic classification, bibliometric analysis can further reveal how research priorities have changed over time and identify areas requiring greater empirical attention.

Although bibliometric approaches have increasingly been applied to agricultural technology and smart farming research, a comprehensive bibliometric assessment specifically focused on smart agriculture research associated with date palm production across the Arabian Peninsula is lacking. This gap is important because the region combines exceptionally severe environmental constraints with a crop of high agricultural and food-security significance. A systematic assessment of the research landscape can therefore provide a basis for understanding the current maturity of the field and for identifying priorities for future research and technology development.

### 1.4. Aim and Objectives of the Study

Against this background, the present study conducts a systematic bibliometric analysis of peer-reviewed research on date palm agriculture and smart agriculture technologies across the Arabian Peninsula and selected adjacent date-producing countries. The analysis covers publications indexed in Scopus and Web of Science between 2016 and 2026 and examines the temporal, geographic, methodological, technological and thematic characteristics of the literature. Specifically, the study aims to:

1. Characterize the evolution and geographic distribution of research on date palm agriculture and smart agriculture in the Arabian Peninsula, including publication trends, contributing countries, journals and international collaboration patterns.
2. Identify the principal research designs, technology categories and thematic domains represented in the literature, with particular attention to remote sensing and UAVs, GIS, IoT and sensors, AI/ML, irrigation technologies and other emerging smart-agriculture approaches.
3. Examine patterns of research impact and collaboration, including citation distributions and the relationship between international collaboration, publication characteristics and scholarly impact.
4. Identify methodological, technological and thematic gaps in the existing literature, particularly with respect to field validation, IoT and smart-farming applications, AI/ML, water management and other emerging research domains.
5. Highlight priorities for future research and technology development that can support the transition toward more efficient, data-driven and sustainable date palm production under the environmental conditions of the Arabian Peninsula.

By integrating bibliometric indicators with technology and topic-based classification, this study provides a quantitative overview of the development, structure and emerging directions of date palm smart-agriculture research. The findings are intended to help researchers identify knowledge gaps, support collaboration and research planning, and provide evidence for the development and evaluation of technologies suited to arid and hyperarid date palm production systems.

## 2. Materials and Methods

### 2.1. Database Selection

**Figure 1:**
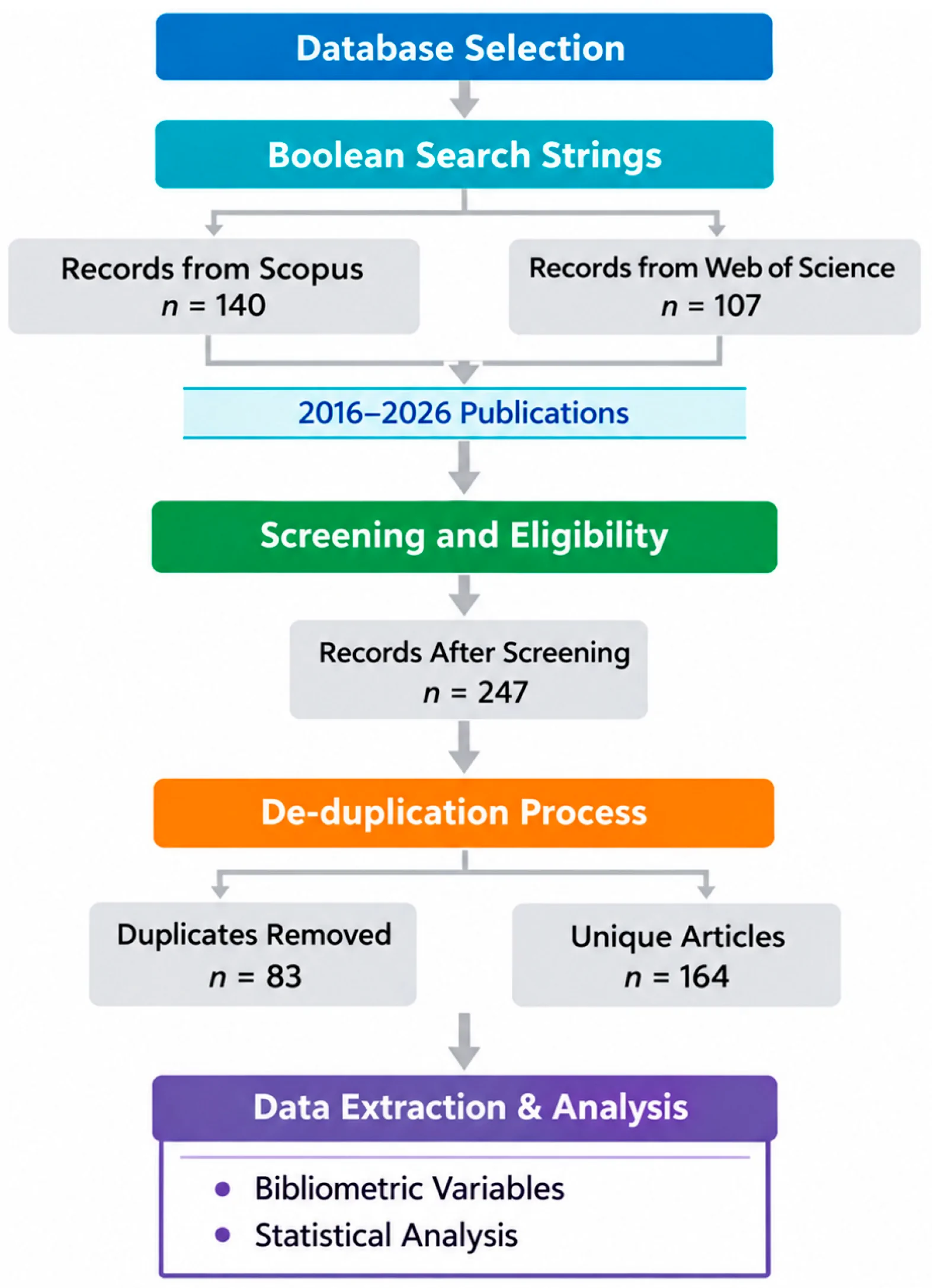
Flow diagram of the literature selection and bibliometric analysis process.

A systematic literature search was conducted to identify peer-reviewed research on smart agriculture technologies applied to date palm (*Phoenix dactylifera* L.) production in the Arabian Peninsula [35]. To ensure comprehensive and high-quality coverage of the scientific literature, two major bibliographic databases, Scopus (Elsevier) and Web of Science (Clarivate Analytics), were selected (Figure 1). These databases were chosen due to their complementary strengths and extensive use in bibliometric studies within agricultural and environmental sciences. Scopus offers broader coverage, particularly of regional and emerging journals, while Web of Science provides more selective indexing, with a strong emphasis on high-impact journals and well-established citation records. The combined use of both databases therefore enhances the completeness, reliability and representativeness of the retrieved literature [36,37].

### 2.2. Boolean Search Strings

Building on the selected databases, a structured search strategy was developed to ensure systematic retrieval of relevant publications. The search was restricted to peer-reviewed journal articles and review papers published in English between 2016 and 2026 (inclusive), thereby capturing recent developments in smart agriculture technologies while maintaining temporal relevance. The year 2016 was selected as the starting point because it corresponds to a period of accelerated development and integration of emerging digital technologies, including the Internet of Things (IoT), artificial intelligence (AI), remote sensing, robotics, and data-driven approaches, within agricultural systems. Limiting the search to this period also helped ensure that the review reflects contemporary technological developments and their recent applications in smart agriculture, while avoiding an excessive inclusion of earlier literature that may have been developed under substantially different technological conditions. The search strings were constructed using Boolean operators and applied consistently across both databases to maximize comparability and reproducibility [38].

Scopus search string:

TITLE-ABS-KEY ((“date palm” OR “*Phoenix dactylifera*”) AND (“Arabian Peninsula” OR “Persian Gulf” OR “Gulf region” OR “GCC” OR “Saudi Arabia” OR “United Arab Emirates” OR “UAE” OR “Oman” OR “Kuwait” OR “Qatar” OR “Bahrain” OR “Iraq” OR “Jordan”) AND (“agriculture” OR “food system” OR “food security” OR “crop production” OR “horticulture” OR “farming systems” OR “food safety” OR “agricultural research” OR “IoT” OR “sensors” OR “biosensors” OR “remote sensing” OR “satellite imagery” OR “GIS” OR “unmanned aerial vehicles” OR “AI” OR “machine learning” OR “big data” OR “DSS”)) AND (DOCTYPE (ar OR re)) AND PUBYEAR > 2015 AND PUBYEAR < 2027 AND (LIMIT-TO (LANGUAGE, “English”)).

Web of Science search string:

TS = ((“date palm” OR “Phoenix dactylifera”) AND (“Arabian Peninsula” OR “Persian Gulf” OR “Gulf region” OR “GCC” OR “Saudi Arabia” OR “United Arab Emirates” OR “UAE” OR “Oman” OR “Kuwait” OR “Qatar” OR “Bahrain” OR “Iraq” OR “Jordan”) AND (“agriculture” OR “food system” OR “food security” OR “crop production” OR “horticulture” OR “farming systems” OR “food safety” OR “agricultural research” OR “IoT” OR “sensors” OR “biosensors” OR “remote sensing” OR “satellite imagery” OR “GIS” OR “unmanned aerial vehicles” OR “AI” OR “machine learning” OR “big data” OR “DSS”)) AND (DT = “Article” OR DT = “Review”) AND LA = “English” AND PY = (2016–2026).

### 2.3. Inclusion and Exclusion Criteria

Following the initial retrieval of records, explicit inclusion and exclusion criteria were applied to ensure a transparent, systematic and reproducible study selection process. These criteria were designed to refine the dataset by retaining only studies that were directly relevant to the research objectives, while excluding irrelevant or low-quality records [39]. The inclusion criteria ensured that all selected articles were peer-reviewed, focused primarily on date palm systems, geographically relevant to the Arabian Peninsula, and aligned with at least one thematic domain related to smart agriculture or associated disciplines. Conversely, the exclusion criteria removed records that did not meet methodological, thematic or geographic requirements, including non-journal publications, studies outside the defined timeframe, and articles in which date palm was not the primary focus (Table 1). Importantly, these criteria were applied sequentially after database retrieval and prior to data processing, thereby ensuring that only relevant and high-quality records were included in the final bibliometric corpus.

### 2.4. Article De-Duplication

The application of the search strategy resulted in two independent datasets: 140 records from Scopus (CSV format) and 107 records from Web of Science (plain text format), yielding a combined total of 247 records. Given the expected overlap between databases, a systematic de-duplication process was required. Both datasets were imported into R (version 4.5.3) using the bibliometrix package, which facilitated format harmonization and metadata standardization across sources [40]. Duplicate detection was performed using a multi-criteria matching approach based on Digital Object Identifiers (DOIs), title similarity (via approximate string matching), and combinations of author, year and journal information. This process identified and removed 83 duplicate records, resulting in a final dataset of 164 unique articles that satisfied all predefined inclusion criteria. The cleaned dataset was exported as a unified data frame, providing a robust foundation for subsequent statistical and thematic analyses.

### 2.5. Bibliometric Variables Extraction

Following data cleaning and de-duplication, a structured variable extraction process was conducted to transform the raw bibliographic records into an analyzable dataset [41]. This step represents a critical bridge between data collection and statistical analysis, as it defines the variables used to characterize the corpus and address the study objectives. A total of 23 bibliometric variables were extracted or derived for each of the 164 articles. The extracted variables formed the analytical backbone of the study, supporting descriptive statistics, inferential testing and thematic mapping (Table 2).

### 2.6. Software

All bibliometric data processing and statistical analyses were conducted using R (version 4.5.3). Data import, cleaning, format harmonization, merging, de-duplication and computation of bibliometric indicators were performed using the bibliometrix package. Inferential statistical analyses were implemented using base R functions [42]. Specifically, linear regression was conducted using lm(), normality testing using shapiro.test(), group comparisons using wilcox.test() (Mann–Whitney U test) and kruskal.test() (Kruskal–Wallis test), and categorical association testing using chisq.test() [43]. Spearman’s rank correlation was computed using cor.test(method = “spearman”). The Mann–Kendall trend test was implemented via a custom R function based on the original formulations of Kendall and Mann, enabling direct computation of the test statistic S, its variance Var(S), the standardized statistic Z, and Kendall’s τ [44]. Data visualization was performed using Python (version 3.14.3), primarily with the matplotlib library and supported by SciPy for additional statistical computations [45]. Graphical outputs included bar charts, line plots and heat maps used to illustrate bibliometric patterns and temporal dynamics.

## 3. Results

### 3.1. Overview of the Literature

The systematic search across Scopus and Web of Science, followed by de-duplication, yielded a final corpus of 164 articles published between 2016 and 2026 (partial year). These articles were distributed across 117 unique journals and originated from 21 countries. The majority of articles (*n* = 150, 91.5%) were classified as original research articles, followed by reviews (*n* = 9, 5.5%), proceedings papers (*n* = 3, 1.8%) and pre-prints (*n* = 2, 1.2%). Open Access articles accounted for 126 of the total corpus (76.8%), indicating a strong trend toward open dissemination of research in this domain. The h-index of the corpus was 23, with a total of 1911 citations and a mean of 11.65 citations per article (SD = 17.77).

The distribution of articles across 117 journals for 164 publications (ratio of 1.40 articles per journal) indicated a remarkably high scatter and low concentration of research output. This pattern is consistent with Bradford’s Law of Scattering, wherein a small core of journals contributes disproportionately to the literature, while the vast majority of journals publish only one or two articles on the topic [46]. Indeed, the top five journals accounted for 29 articles (17.7%), whereas the remaining 112 journals published fewer than four articles each. The Herfindahl concentration index for this distribution was exceedingly low, confirming the absence of a dominant publication venue and reflecting the multidisciplinary nature of date palm smart agriculture research [47]. International collaboration was observed in 62 articles (37.8%), while contributions from GCC countries accounted for 93 articles (56.7%). Table 3 presents a summary of the key descriptive statistics of the reviewed corpus.

### 3.2. Publication Trend Analysis

**Figure 2:**
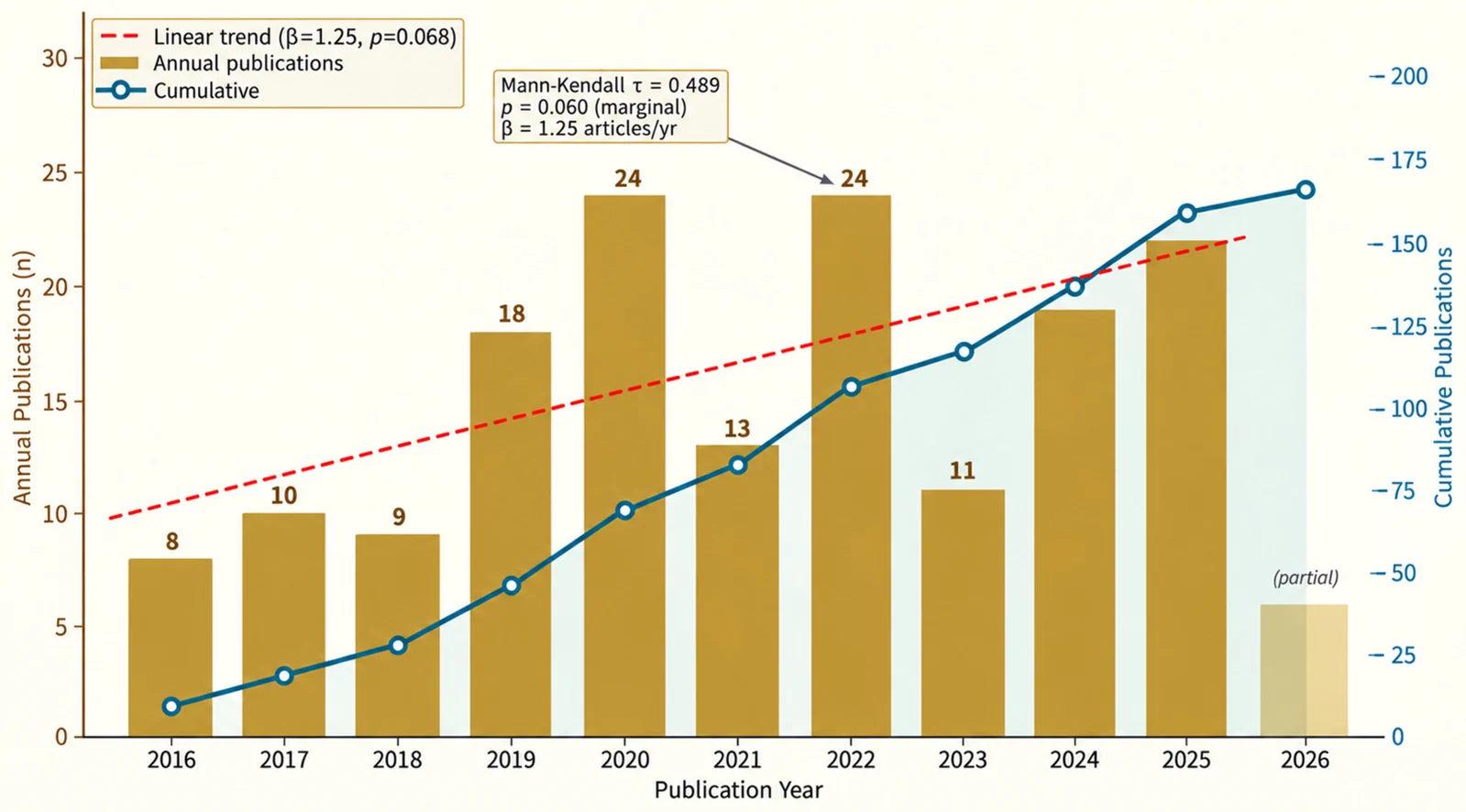
Annual and cumulative publication trend on date palm agriculture across Arabian Peninsula.

The annual distribution of publications revealed a general upward trajectory over the study period, with output increasing from 8 articles in 2016 to 22 articles in 2025, representing a 175% increase and an approximate annual growth rate of 10.9% (Figure 2). Two distinct peaks were observed in 2020 and 2022, each yielding 24 articles. A notable dip occurred in 2021 (*n* = 13), which may reflect publication delays attributable to the COVID–19 pandemic and associated disruptions to research activities and journal processing timelines. The subsequent recovery in 2022 (*n* = 24) suggested a rebound effect, potentially augmented by the backlog of manuscripts delayed during the pandemic period [49].

Linear regression analysis of the annual publication counts yielded a positive slope of β = 1.25 articles per year, although the fit was modest (R^2^ = 0.358) and the trend did not reach conventional statistical significance (*p* = 0.068). The non-parametric Mann–Kendall trend test corroborated this finding, producing a marginally significant positive trend (τ = 0.489, z = 1.878, *p* = 0.060). Although neither test achieved significance at the α = 0.05 threshold, both indicated a consistent directional increase in research output over the decade. The cumulative publication trajectory reached 164 articles by mid-2026, with the partial 2026 count (*n* = 6) suggesting continued growth. The year-by-year counts were as follows: 2016 (*n* = 8), 2017 (*n* = 10), 2018 (*n* = 9), 2019 (*n* = 18), 2020 (*n* = 24), 2021 (*n* = 13), 2022 (*n* = 24), 2023 (*n* = 11), 2024 (*n* = 19), 2025 (*n* = 22) and 2026 (*n* = 6, partial).

### 3.3. Geographic Distribution and International Collaboration

**Figure 3:**
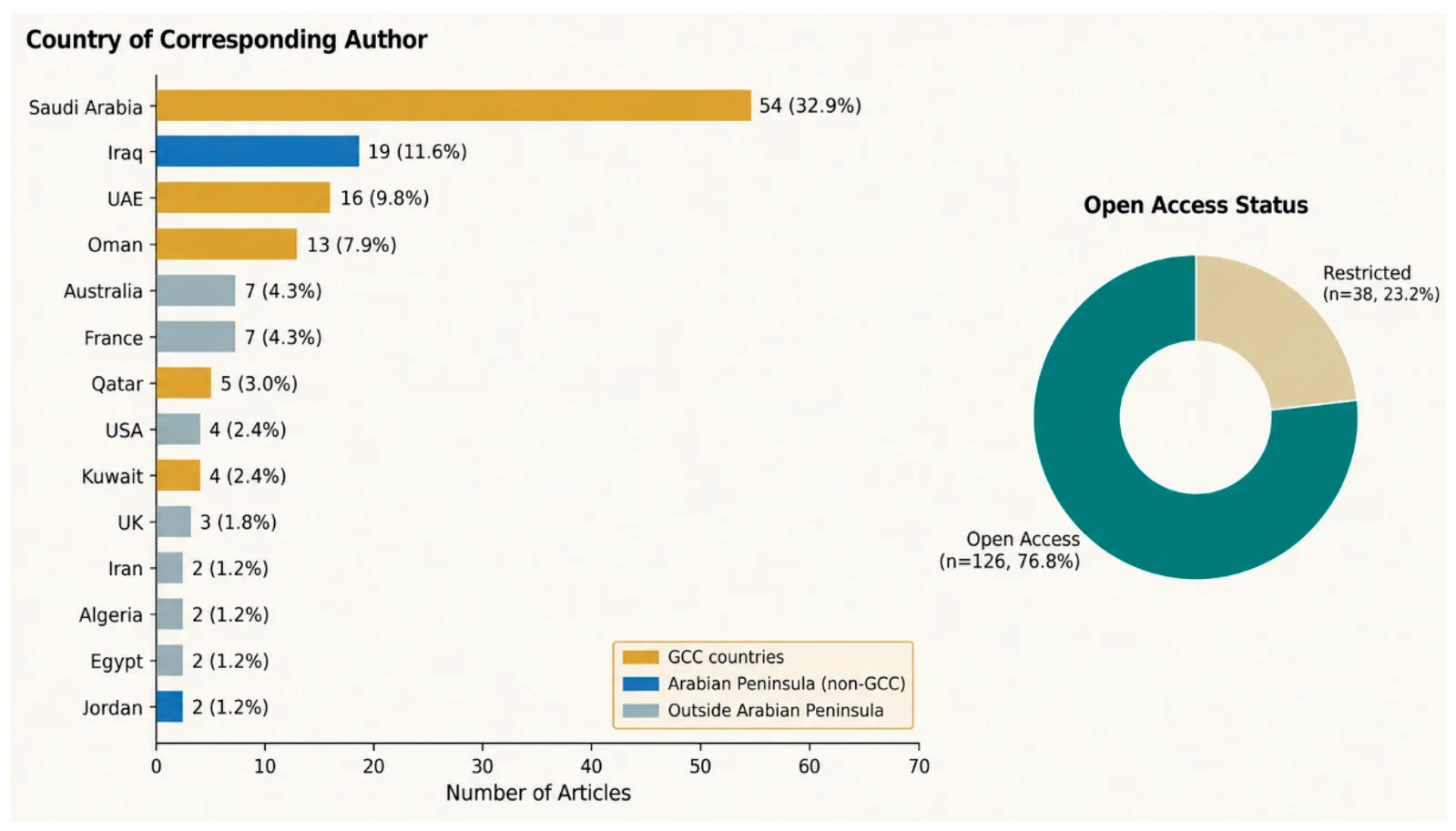
Geographic distribution of articles by country of corresponding author and open access status.

The geographic analysis revealed a pronounced concentration of research output in the GCC countries, which collectively contributed 92 articles (56.7%) to the corpus (Figure 3). Saudi Arabia was the leading contributor with 54 articles (32.9%), followed by the United Arab Emirates (*n* = 16, 9.8%), Oman (*n* = 13, 7.9%), Qatar (*n* = 5, 3.0%) and Kuwait (*n* = 4, 2.4%). Non GCC Arabian Peninsula countries (Iraq and Jordan) contributed 21 articles (12.8%), while researchers based outside the Arabian Peninsula accounted for 51 articles (30.5%), with notable contributions from Australia (*n* = 7, 4.3%), France (*n* = 7, 4.3%) and the United States (*n* = 4, 2.4%). This geographic distribution reflects the regional centrality of date palm cultivation and associated smart agriculture research priorities. The dominance of Saudi Arabia aligns with its substantial investment in agricultural technology and date palm production as part of its Vision 2030 diversification strategy [50].

**Figure 4:**
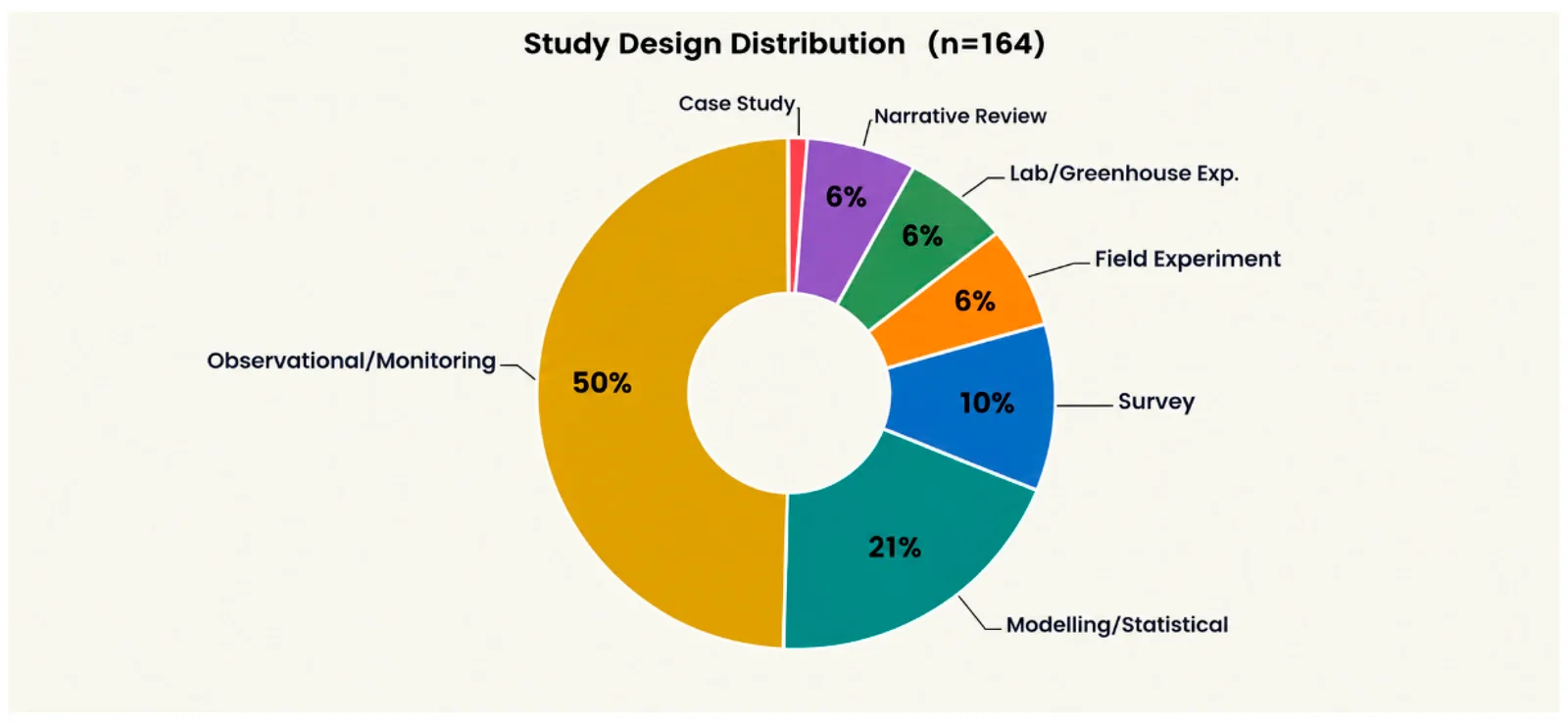
Distribution of document types and study designs in the bibliometric corpus.

International collaboration was identified in 62 articles (37.8%) of the corpus. The collaboration rate varied substantially by region: researchers based outside the Arabian Peninsula exhibited the highest international collaboration rate (56.0%), whereas GCC based researchers collaborated internationally in 34.4% of their publications and non GCC Arabian Peninsula researchers demonstrated the lowest rate (9.5%) (Table 3). A chi-square test revealed a statistically significant association between international collaboration and Open Access publication status (χ^2^ = 4.11, *p* = 0.043), suggesting that internationally collaborative research was more likely to be published in Open Access venues (Figure 4). Critically, international collaboration was associated with substantially higher citation impact. Articles involving international collaboration received a mean of 16.55 citations compared to 8.68 citations for domestically authored articles and this difference was highly statistically significant (Mann–Whitney U test, *p* = 0.0007). This finding underscores the importance of cross border research partnerships in enhancing the visibility and impact of date palm research [51].

### 3.4. Document Types and Study Designs

Original research articles dominated the corpus (*n* = 150, 91.5%), with review papers constituting only 5.5% (*n* = 9) of the total output. The relatively low number of review articles suggests that the field remains at a stage where primary data generation is prioritized over synthesis and consolidation. Narrative reviews accounted for three articles (1.8%), while case studies were the least common design (*n* = 2, 1.2%). This distribution further supports the observation that the research landscape is still maturing, with limited efforts to aggregate or critically appraise the existing evidence base. The strong preference for original research indicates that researchers are actively building foundational knowledge rather than synthesizing established findings.

Building directly on this pattern of empirical primacy, the analysis of study designs confirms that observational approaches dominate the methodological landscape. Observational and monitoring studies were the most prevalent methodology, accounting for 82 articles (50.0%). Modeling and statistical approaches represented the second most common design (*n* = 34, 20.7%), followed by surveys (*n* = 16, 9.8%). Notably, field experiments (*n* = 10, 6.1%) and laboratory/greenhouse experiments (*n* = 10, 6.1%) together comprised only 12.2% of the corpus (Figure 4). The low proportion of experimental studies, particularly field experiments, represents a significant methodological gap in the literature. This gap is especially critical because field experiments provide the strongest evidence for causal claims regarding smart agriculture interventions under real-world conditions. Without such experimental validation, the scalability and effectiveness of technologies like IoT-based irrigation or remote sensing remain uncertain. The predominance of observational designs may limit the ability to establish causal relationships between smart agriculture interventions and date palm production outcomes [52].

### 3.5. Citation Analysis and Research Impact

**Figure 5:**
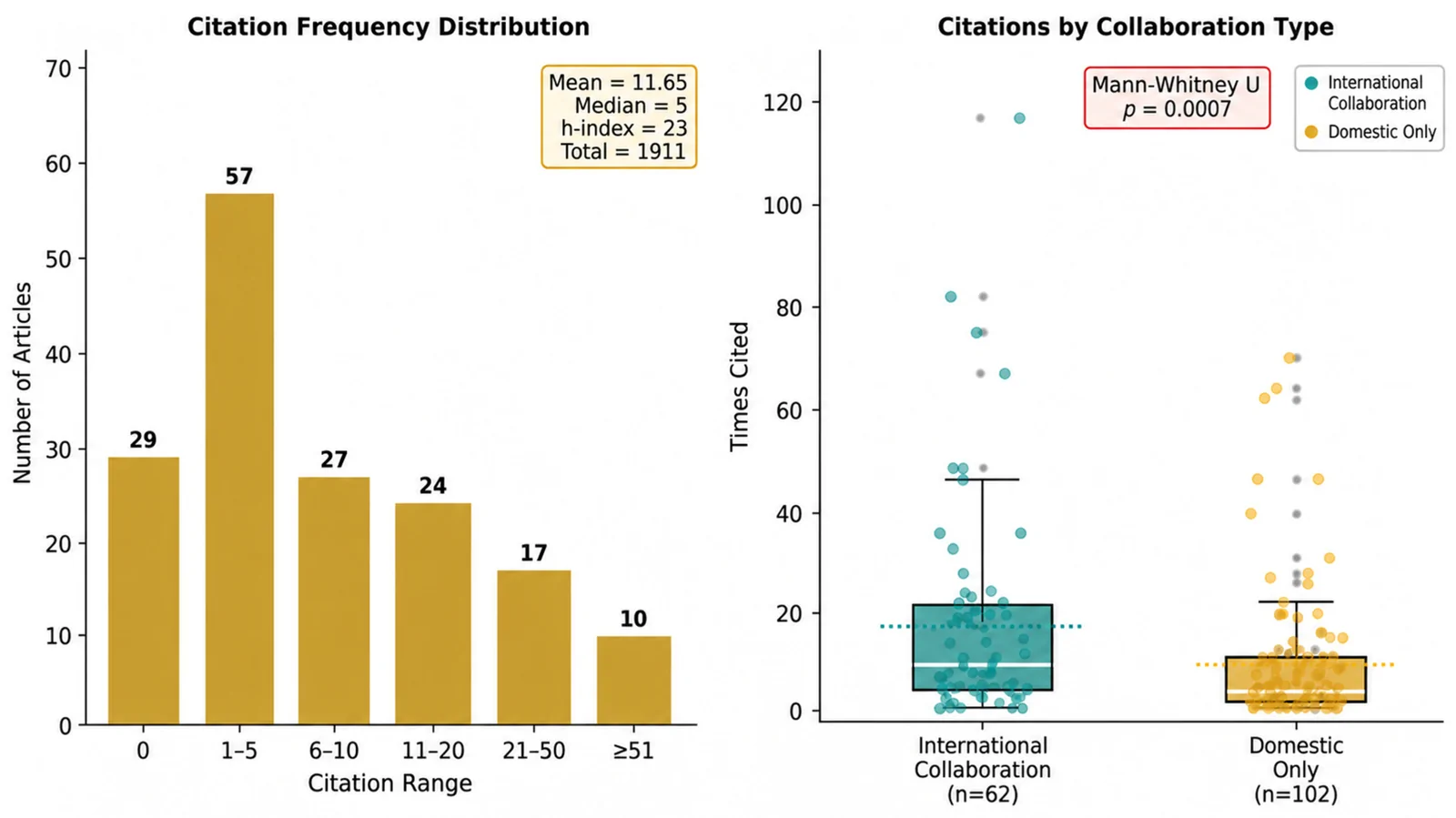
Citation frequency distribution and comparison of citation impact between internationally collaborative and domestic only studies.

The 164 articles in the corpus accumulated a total of 1911 citations, yielding a mean of 11.65 citations per article (SD = 17.77) and a median of five citations. The corpus-level h-index was 23, indicating that 23 articles received at least 23 citations each. The citation distribution was highly right-skewed, with 29 articles (17.7%) remaining uncited and the majority of articles (approximately 57 articles) receiving between one and five citations. Twenty-seven articles (16.5%) were classified as highly cited, having received 20 or more citations. The most cited article in the corpus was that of [53] with 165 citations (Table 4). This skewed distribution is typical of emerging research fields, where a small number of influential studies attract disproportionate attention while many contributions remain under cited. The presence of nearly 18% uncited articles further suggests that a substantial portion of the literature has yet to gain traction within the research community. The Spearman rank correlation between publication year and citation count was negative, consistent with the expected pattern wherein more recent articles have had less time to accumulate citations (Figure 5). Thus, while temporal factors partially explain citation variability, the skewed distribution also points to underlying differences in research quality, relevance or visibility across the corpus.

Extending this citation analysis to examine potential drivers of impact, the effect of international collaboration on citation impact was statistically significant: internationally collaborative articles received a mean of 16.55 citations versus 8.68 for domestically authored articles (Mann–Whitney U test, *p* = 0.0007). This finding reinforces the importance of cross border partnerships in enhancing research visibility and scholarly influence within the date palm smart agriculture domain. In contrast, Open Access status did not significantly influence citation counts (Open Access mean = 12.04 vs. non-OA mean = 10.37; Mann–Whitney U test, *p* = 0.643). This result indicates that while Open Access facilitates broader dissemination, it does not independently drive citation accumulation in this field, and that the quality and collaborative breadth of the research are more important determinants of impact [63].

Complementing the quantitative citation analysis with a qualitative examination of high impact articles, Table 4 presents the ten most cited articles in the corpus. The geographic distribution of these highimpact works spans three nations: the United Arab Emirates (UAE), the United Kingdom, and Saudi Arabia. Notably, the UAE dominates this list, securing seven of the top ten positions. This contrasts sharply with Saudi Arabia’s leading role in overall publication volume (32.9% of the corpus), appearing only once in the top ten. This disconnect underscores a crucial distinction in the field: a high volume of research output does not automatically equate to outsized scholarly influence [64].

### 3.6. Citation Analysis by Technology Categories

**Figure 6:**
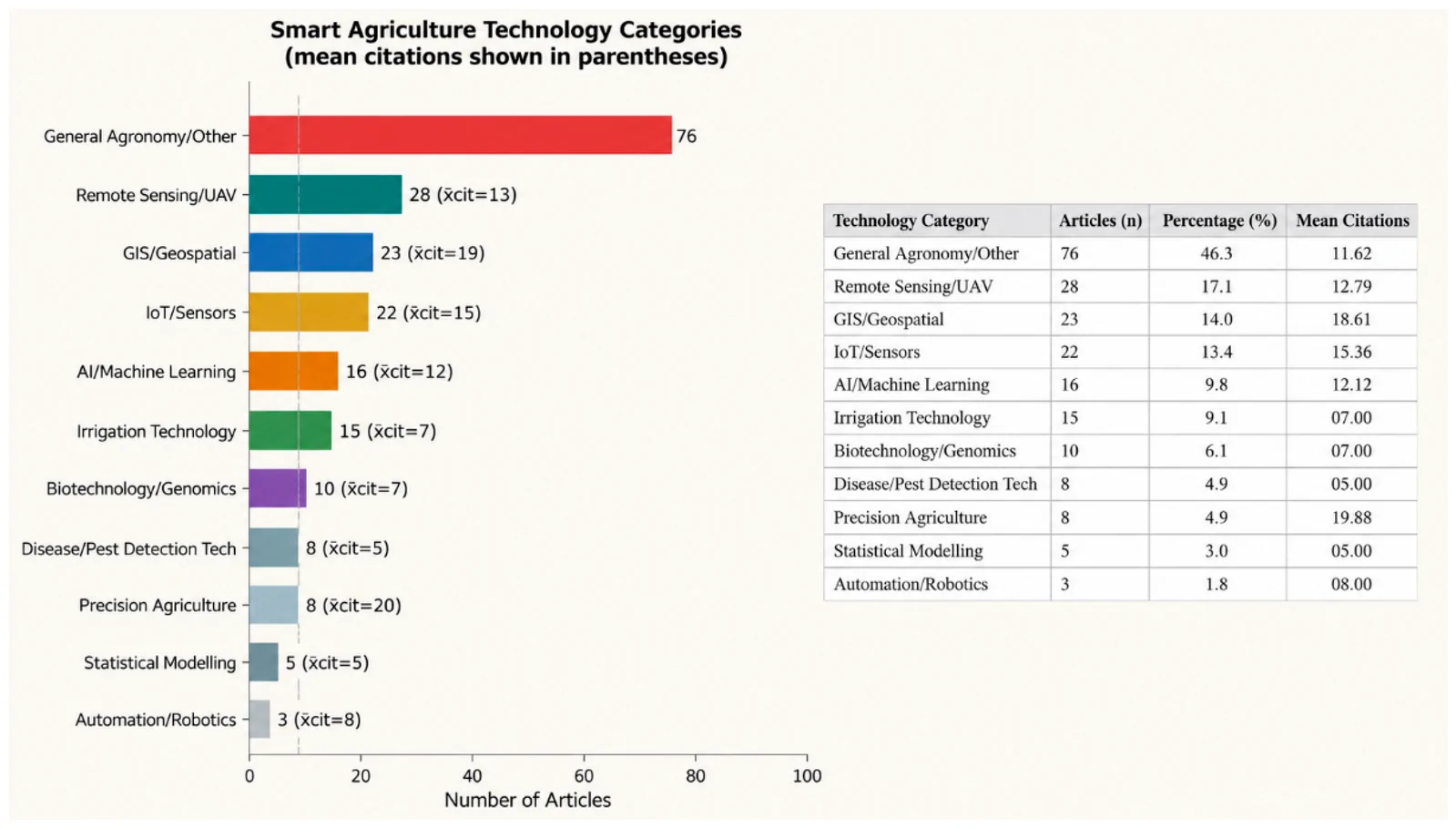
Technology category distribution and mean citation impact in the bibliometric corpus.

Articles were coded into 11 non-mutually exclusive technology categories to capture the breadth of smart agriculture approaches applied to date palm research (Figure 6). The General Agronomy/Other category, which encompassed studies not employing a specific smart agriculture technology, was the most prevalent (*n* = 76, 46.3%), indicating that nearly half of the literature on date palm production in the Arabian Peninsula does not yet incorporate advanced technological approaches. Moving from the prevalence of technology categories to their scholarly impact, among the smart agriculture technologies, Remote Sensing and Unmanned Aerial Vehicle (UAV) applications were the most widely adopted (*n* = 28, 17.1%), followed by GIS/Geospatial technologies (*n* = 23, 14.0%), IoT/Sensors (*n* = 22, 13.4%) and AI/Machine Learning (*n* = 16, 9.8%). Irrigation technology was addressed in 15 articles (9.1%) and Biotechnology/Genomics in 10 articles (6.1%). Disease/Pest Detection Technology (*n* = 8, 4.9%), Precision Agriculture (*n* = 8, 4.9%), Statistical Modeling (*n* = 5, 3.0%) and Automation/Robotics (*n* = 3, 1.8%) were the least represented categories.

Building directly on the technology distribution, citation analysis revealed variation in scholarly attention across technology categories. Precision Agriculture articles had the highest mean citation count (19.88), followed by GIS/Geospatial (18.61), IoT/Sensors (15.36), Remote Sensing/UAV (12.79), and AI/Machine Learning (12.12), while General Agronomy/Other articles had a mean of 11.62 citations. These differences describe variation in citation impact within the indexed literature and should not be interpreted as evidence that one technology is technically more effective or mature than another. In particular, the relatively high mean citation count for Precision Agriculture was based on only eight articles, whereas Remote Sensing/UAV comprised 28 articles. Differences in publication year, study topic, journal, citation accumulation time, and sample size may also contribute to variation in citation counts. Accordingly, Figure 6 is interpreted as a bibliometric comparison of research activity and scholarly attention rather than a measure of technological performance.

**Figure 7:**
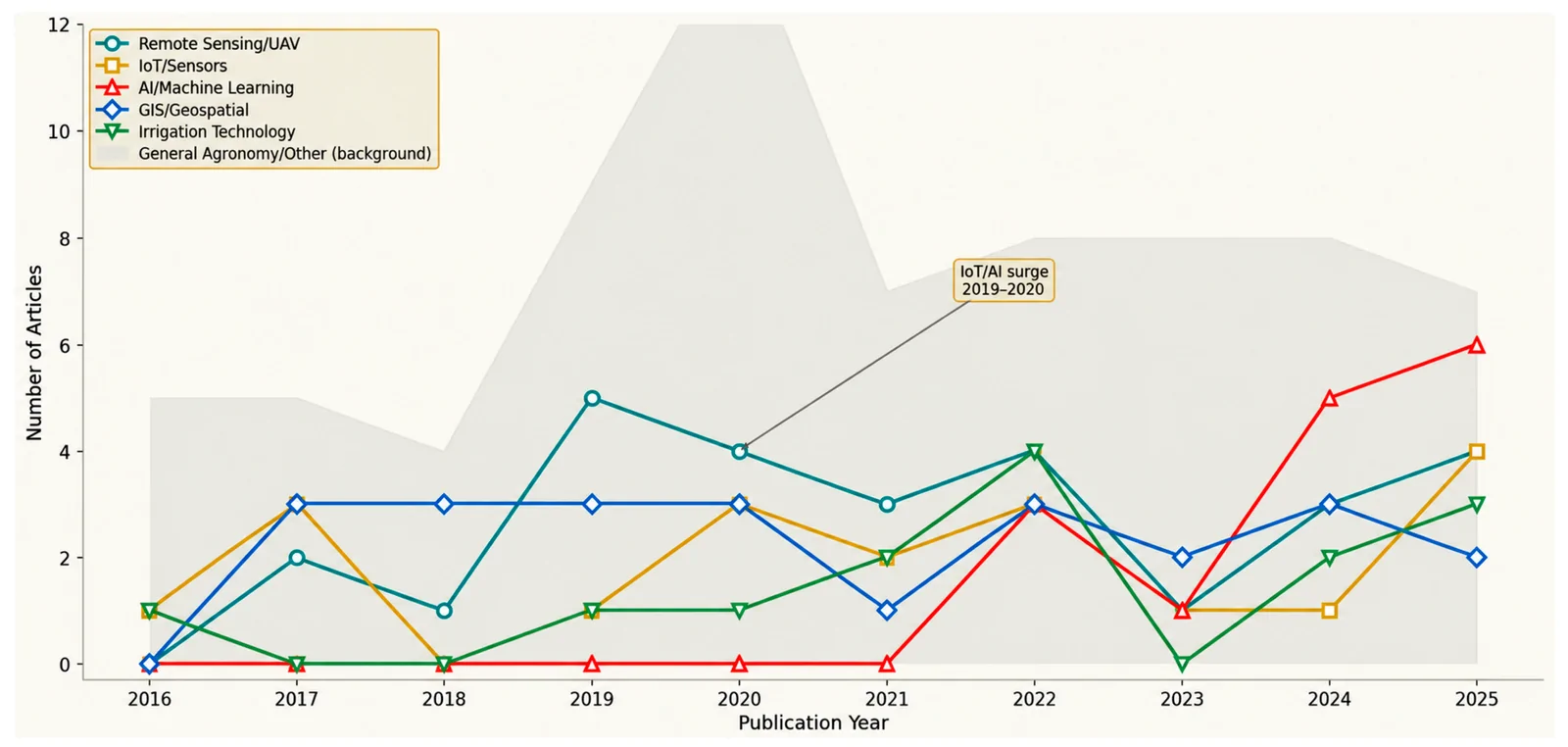
Temporal adoption trends in key smart agriculture technologies for date palm (2016–2026).

Extending the analysis beyond citation impact to examine geographic and temporal patterns, a chi-square test of independence between technology category and geographic region yielded a statistically significant result (χ^2^ = 29.4, *p* = 0.039), indicating that the adoption of specific technologies varied significantly across regions. This regional variation implies that local priorities, resource availability, and institutional expertise shape technology adoption patterns in date palm smart agriculture. For instance, water scarce regions may prioritize irrigation technologies, while pest prone areas may focus on disease detection systems. Temporal analysis revealed that AI/Machine Learning research showed the strongest recent increase, rising from no identified articles before 2023 to six articles during 2023–2025 (Figure 7). This pattern indicates increasing research attention to AI/ML applications within the date palm literature and identifies AI/ML as an emerging component of the research landscape. However, the observed increase reflects publication activity and should not be interpreted as evidence of technological superiority or readiness for widespread field deployment [65].

It is important to emphasize that technology frequency and citation impact in this bibliometric analysis represent levels of research activity and scholarly attention rather than direct measures of technological effectiveness, maturity, or readiness for practical deployment. The classification identifies which smart-agriculture technologies have been investigated in date palm research and how their research presence has evolved across time and geographic contexts. Detailed assessment of sensor performance, algorithmic accuracy, field validation, environmental robustness, economic feasibility, infrastructure requirements, and technology-readiness levels would require a separate technology-focused systematic review or meta-analysis and was therefore beyond the scope of the present study. The present findings should consequently be interpreted as a mapping of the research and technology landscape rather than as a comparative ranking of technological performance.

### 3.7. Research Topic Analysis

**Figure 8:**
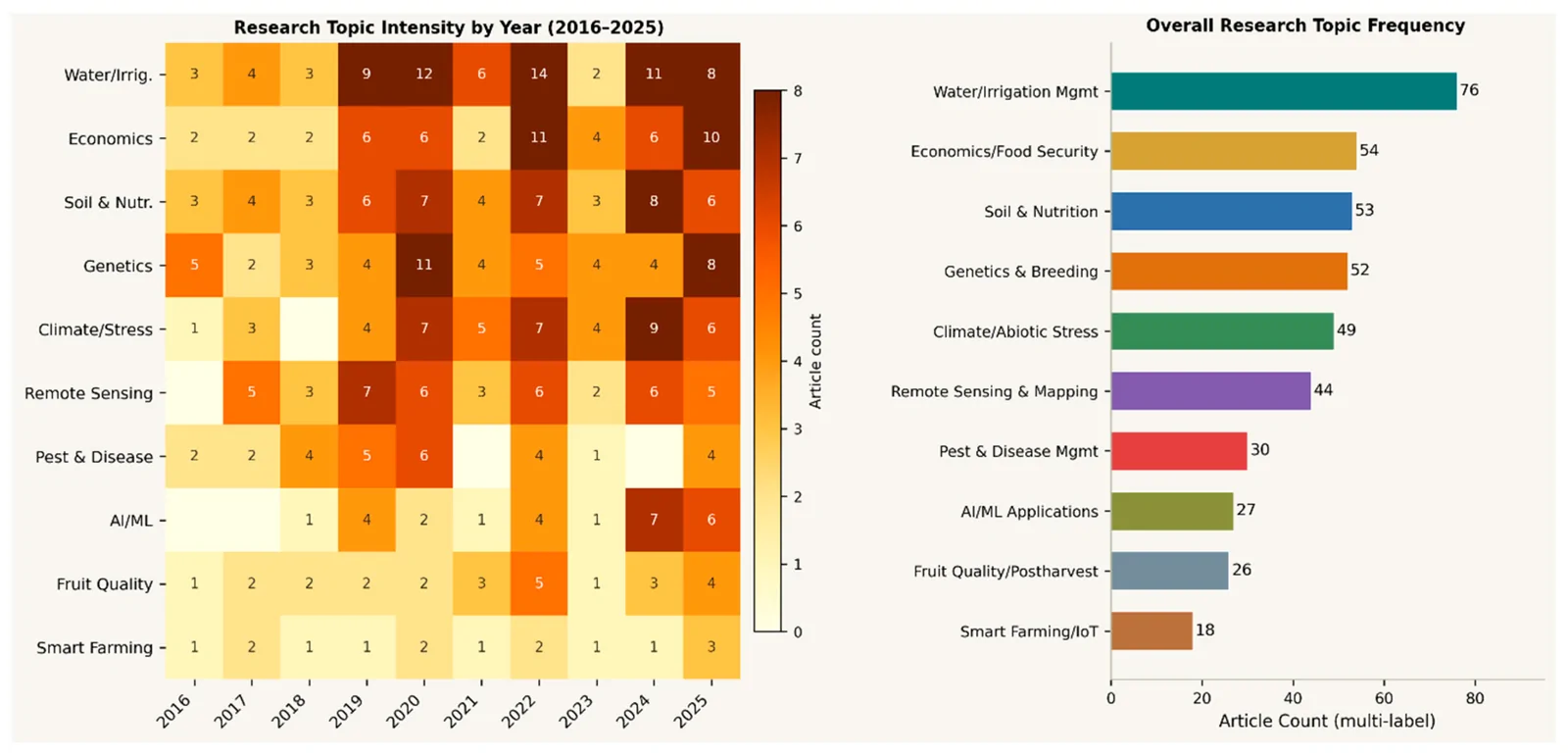
Research topic intensity heat map by year and overall topic frequency distribution.

Multi-label topic coding identified ten major research domains across the corpus (Figure 8). Water and Irrigation Management was the most frequently addressed topic (*n* = 76), reflecting the centrality of water scarcity as a challenge for date palm cultivation in the Arabian Peninsula. Economics and Food Security (*n* = 54) and Genetics and Breeding (*n* = 52) constituted the next largest thematic clusters, underscoring the dual emphasis on economic sustainability and varietal improvement in the region’s date palm research agenda. Soil and Nutrition research was addressed in 53 articles, while Climate and Abiotic Stress appeared in 49 articles, indicating substantial concern with the environmental pressures facing date palm cultivation under changing climatic conditions. Remote Sensing and Mapping (*n* = 44) and Pest and Disease Management (*n* = 30) represented moderately studied topics. AI/ML Applications (*n* = 27) and Fruit Quality/Postharvest (*n* = 26) were emerging areas, while Smart Farming/IoT remained the least studied topic domain (*n* = 18, 11.0%), representing a notable gap given the increasing global emphasis on digitally enabled precision agriculture. A temporal heat map analysis further revealed an intensification of research on Water Management and AI/ML Applications in the post-2022 period, suggesting a shift toward technology enabled solutions for the region’s most pressing agricultural challenges.

### 3.8. Journal Analysis

**Figure 9:**
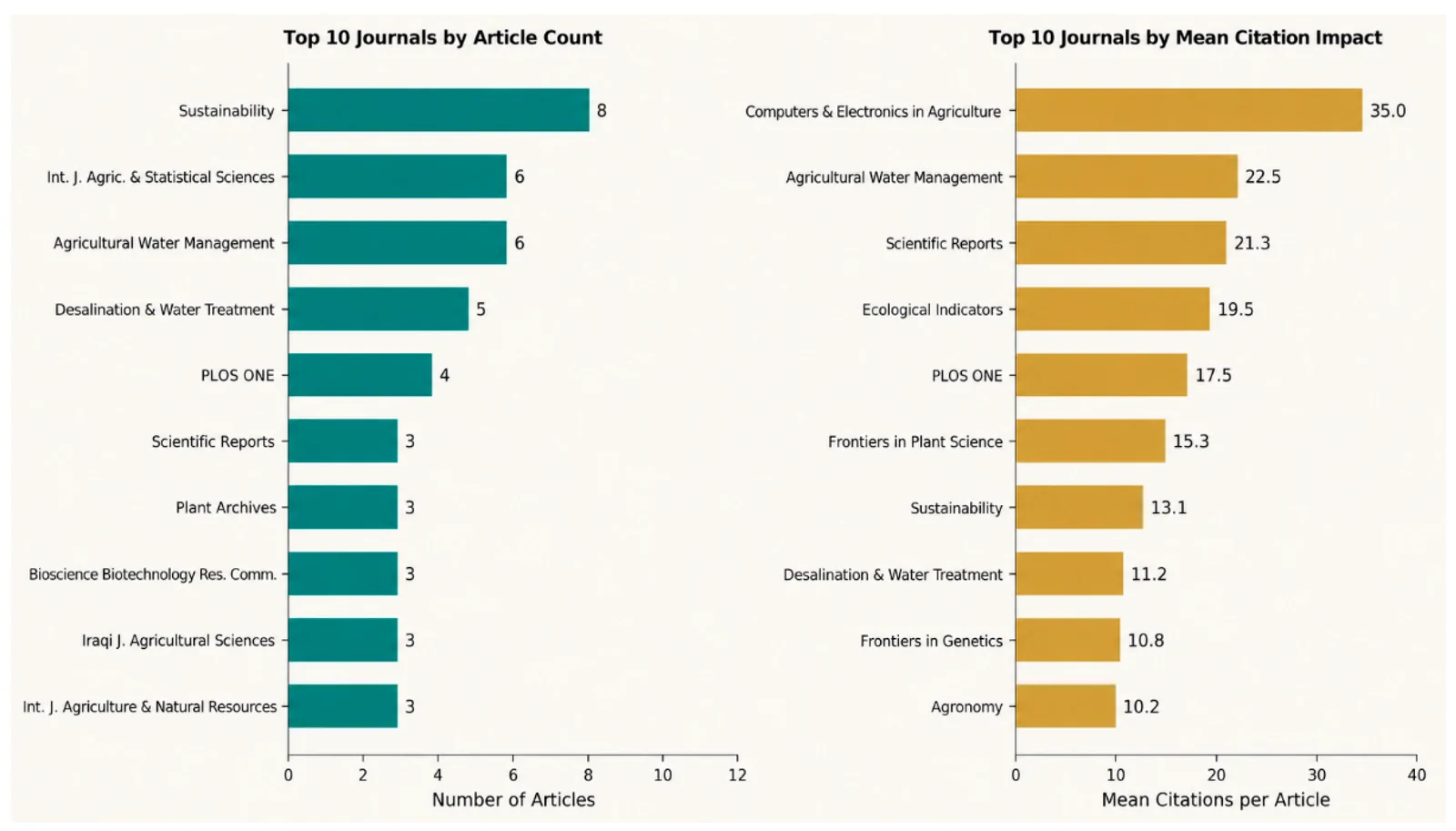
Top contributing journals ranked by article frequency and mean citation impact.

The 164 articles were distributed across 117 unique journals, yielding a highly dispersed publication landscape (Figure 9). The journal Sustainability was the most prolific venue with 8 articles (4.9%), followed by Agricultural Water Management (*n* = 6, 3.7%), International Journal of Agricultural and Statistical Sciences (*n* = 6, 3.7%), Desalination and Water Treatment (*n* = 5, 3.0%) and PLOS ONE (*n* = 4, 2.4%). The Herfindahl concentration index for journal output was exceedingly low, confirming the multidisciplinary and fragmented nature of the publication landscape. While the distribution of articles across journals reveals where research is being published, a more instructive perspective emerges when examining which journals achieve the greatest scholarly influence. An important discrepancy was observed between journal prolificacy and citation impact. When journals were ranked by mean citation impact, Computers and Electronics in Agriculture achieved the highest mean of 35.0 citations per article, followed by Agricultural Water Management (22.5), Scientific Reports (21.3), Ecological Indicators (19.5) and PLOS ONE (17.5). Notably, Sustainability, despite being the most prolific journal, was not among the top five by citation impact. This finding directly contrasts quantity with quality: publishing many articles on a topic does not guarantee that those articles will be widely cited or influential. This discrepancy suggests that the most productive venues for this research area are not necessarily the most impactful [66].

### 3.9. Keyword Analysis and Thematic Clusters

**Figure 10:**
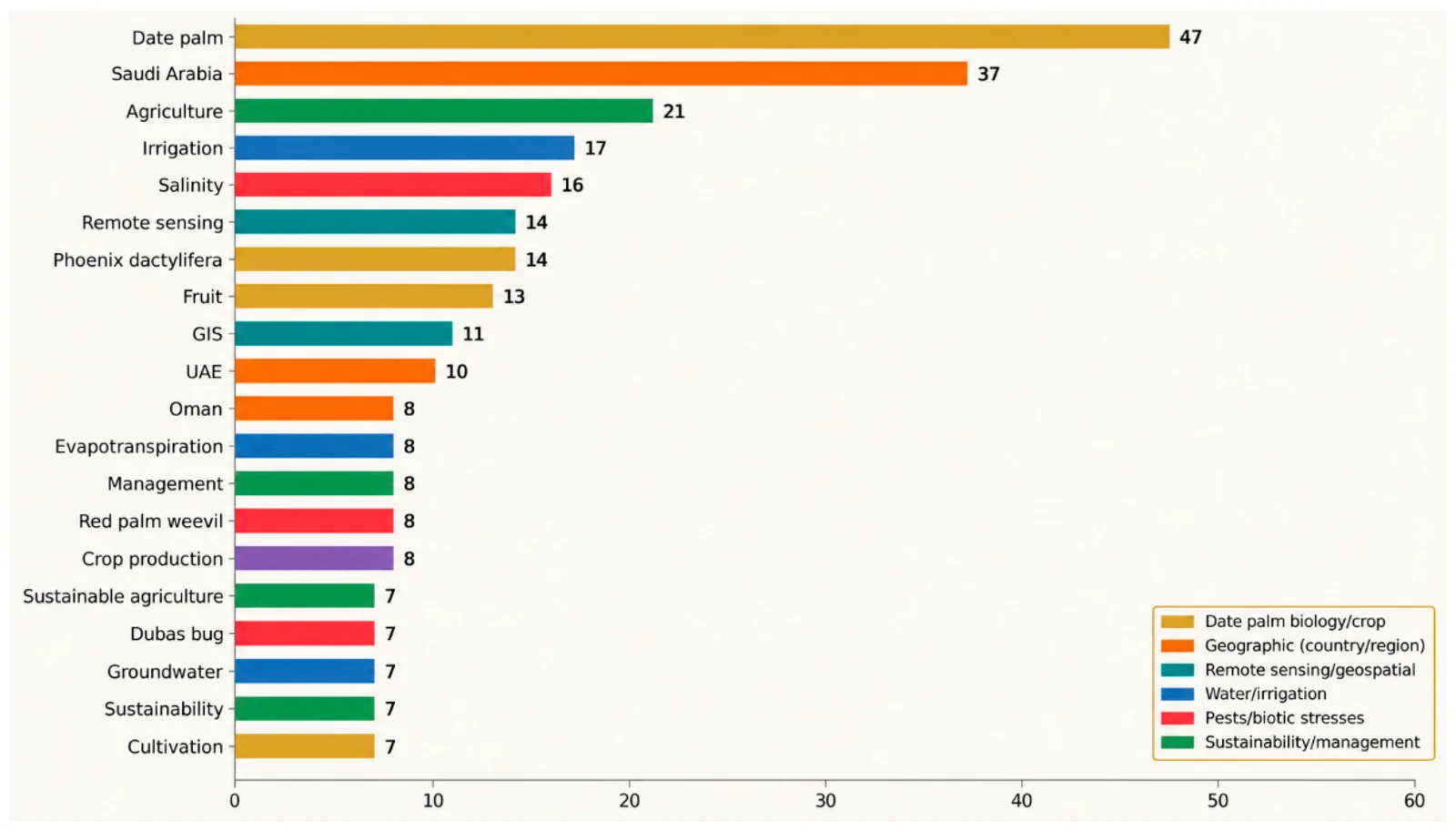
Top twenty keywords by combined frequency (author + index keywords).

Author keyword analysis identified several distinct thematic clusters within the corpus (Figure 10). The keyword date palm was the most frequently occurring term (*n* = 47), followed by Saudi Arabia (*n* = 37), agriculture (*n* = 21), irrigation (*n* = 17), salinity (*n* = 16), remote sensing (*n* = 14), *Phoenix dactylifera* L. (*n* = 14), fruit (*n* = 13), GIS (*n* = 11) and UAE (*n* = 10). The prominence of Saudi Arabia among the top keywords reflects the country’s dominant contribution to the corpus (54 articles, 32.9%), while the presence of UAE and salinity underscores the region’s specific geographic and environmental research priorities. The inclusion of both the common name date palm and its scientific binomial *Phoenix dactylifera* L. indicates consistent taxonomic precision across the literature.

**Figure 11:**
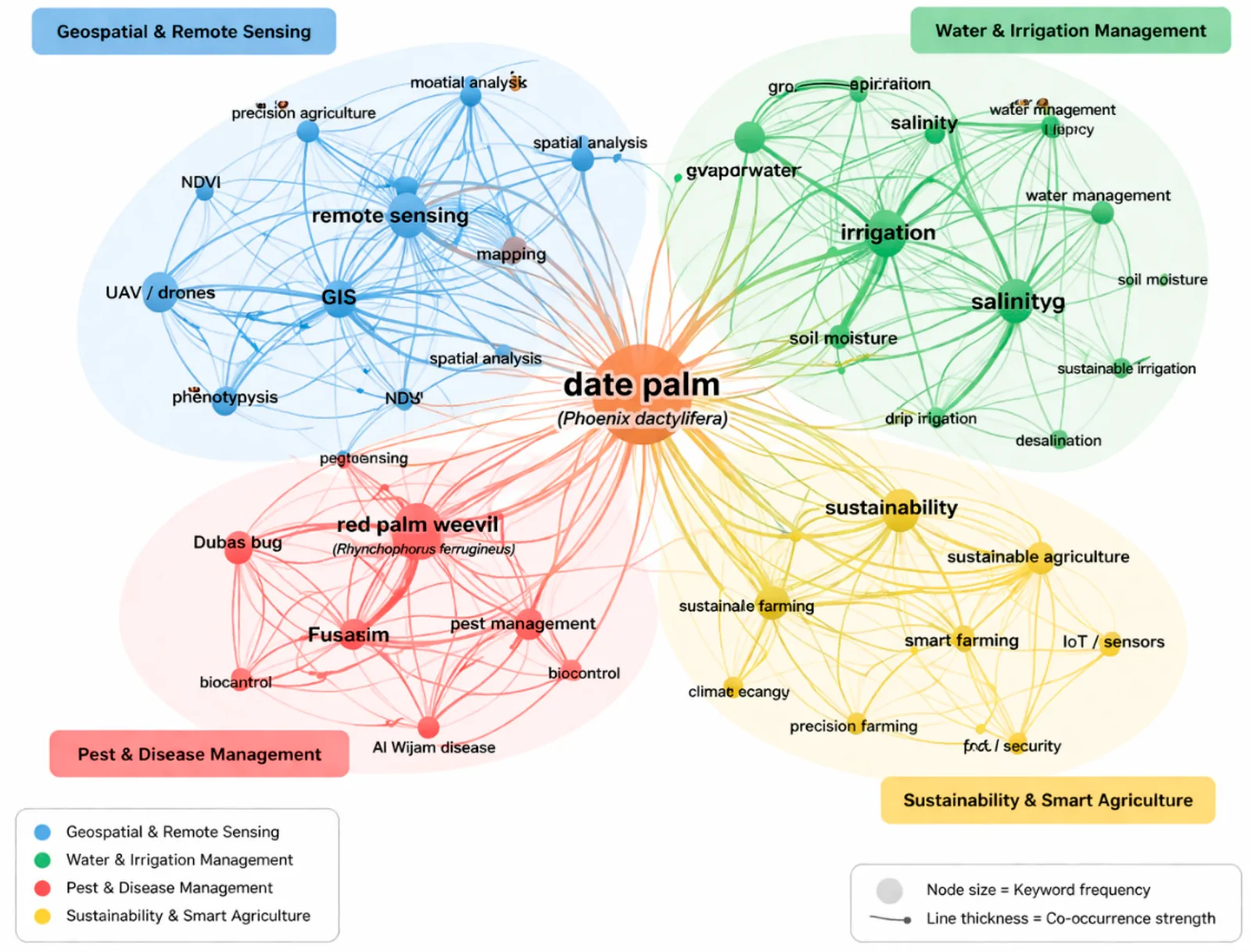
Keyword co-occurrence network and thematic clusters in date palm research (2016–2026).

Extending the analysis from keyword frequency to relational patterns, co-occurrence network analysis revealed four principal thematic clusters. First, a geotechnical cluster centered on remote sensing, GIS, UAV, vegetation indices and NDVI, reflecting the substantial body of work employing earth observation technologies for date palm monitoring and mapping. This cluster directly aligns with the earlier finding that Remote Sensing and UAV applications were the most widely adopted smart agriculture technologies (17.1% of the corpus). Second, a water management cluster comprising keywords such as irrigation, salinity, evapotranspiration and groundwater, which aligned with the dominant research topic of Water and Irrigation Management identified in the topic analysis. The presence of salinity among both the top individual keywords and this cluster confirms that water quality issues are as critical as water quantity in regional date palm research. Third, a pest management cluster including red palm weevil (*n* = 8), Dubas bug (*n* = 7) and *Rhynchophorus ferrugineus*, indicating a focused research effort on the two most economically significant pests of date palms in the region. Notably, pest related keywords did not appear prominently in the high frequency list, yet their strong co-occurrence network reveals a concentrated research community addressing this applied challenge. Fourth, an emerging sustainability cluster characterized by keywords such as sustainability and sustainable agriculture, which appeared predominantly in publications from recent years (2023–2025), suggesting an evolving research agenda that increasingly frames date palm production within broader sustainability and food security narratives (Figure 11).

## 4. Discussion

The present systematic bibliometric analysis provides the first comprehensive mapping of smart agriculture research applied to date palm (*Phoenix dactylifera* L.) production across the Arabian Peninsula over the decade 2016–2026. By synthesizing 164 peer-reviewed articles identified through a string-guided search of Scopus and Web of Science, this review has revealed a research landscape characterized by modest but growing output, significant geographic concentration, a persistent technology gap and emerging methodological innovations. These findings collectively define the current state and future trajectory of digital transformation in one of the world’s most climatically extreme agricultural systems.

The temporal trajectory of publication output reveals a field experiencing gradual expansion, with annual production increasing from eight articles in 2016 to twenty two in 2025, representing a 175% growth over the decade. Although the Mann–Kendall trend test yielded only marginally significant results (τ = 0.489, *p* = 0.060), the consistency between parametric and non-parametric approaches provides convergent evidence of a positive directional trend. This pattern aligns with a broader bibliometric analysis of agricultural technology adoption in the Middle East and North Africa region, where research output on precision agriculture has similarly shown steady but not explosive growth over the past decade [67,68].

However, when compared to major global commodity crops such as wheat, maize and rice, the volume of smart agriculture research dedicated to date palm remains disproportionately low [69]. Previous research identified more than 3000 articles on smart farming technologies applied to cereal crops over a similar period, underscoring the relative neglect of tree crops, particularly those confined to arid regions, in the digital agriculture revolution. This disparity reflects what the present analysis identifies as a persistent technology gap, wherein the foundational technologies for digital transformation are being developed and tested in other cropping systems but have yet to be adapted, validated and scaled for date palm production.

The geographic distribution of research output reveals a pronounced concentration in the GCC countries, with Saudi Arabia alone contributing nearly one-third of all articles in the corpus. This pattern reflects the substantial investments that GCC nations have made in agricultural research and development as part of their national food security strategies. Saudi Arabia’s Vision 2030 and the UAE’s National Food Security Strategy 2051 have explicitly prioritized agricultural innovation and technology adoption, creating institutional frameworks that incentivize research on smart agriculture solutions. The dominance of Saudi Arabia is consistent with its position as the world’s second-largest date producer and its substantial investment in date palm research infrastructure [70].

A critical finding concerns the role of international collaboration in enhancing research impact. Articles involving international collaboration received a mean of 16.55 citations compared to 8.68 for domestically authored articles (*p* = 0.0007), representing a nearly twofold increase in scholarly attention. This finding is consistent with extensive bibliometric literature demonstrating that internationally collaborative research consistently achieves higher citation counts across scientific disciplines [71]. In the context of date palm smart agriculture, the benefits of such collaboration are particularly pronounced given the specialized expertise required to integrate advanced technologies with domain specific knowledge of date palm physiology and Arabian Peninsula agro-ecosystems. Notably, the top ten most cited articles originated from three different countries, demonstrating that high impact research in this field is increasingly conducted through international partnerships.

The analysis of study designs reveals a methodological landscape dominated by observational and monitoring studies, which together accounted for 50% of the corpus, while field experiments represented only 6.1% of all articles. This methodological imbalance has important implications for the validity and generalization of the evidence base. Observational studies, while valuable for describing patterns and generating hypotheses, are inherently limited in their ability to establish causal relationships between smart agriculture interventions and date palm production outcomes.

This pattern is not unique to date palm research, [72] noted a similar predominance of proof-of-concept studies relative to rigorous field trials across the broader agricultural technology literature. However, the gap may be particularly consequential for date palm systems, where the long generation time of the crop (5–8 years to reach full productivity), the spatial heterogeneity of oasis agro-ecosystems and the extreme environmental conditions demand robust experimental validation. The low proportion of field experiments also reflects practical challenges, including the large land area required for replicated trials and the difficulty of controlling environmental variables in open field conditions. As [73] emphasized, the lack of long-term field experiments represents a major obstacle to developing evidence-based management recommendations, a finding that aligns with the present analysis’s identification of field experiments as the least represented study design.

The technology category analysis reveals that nearly half of the research corpus (46.3%) falls within the General Agronomy/Other category, indicating that smart agriculture technologies have not yet become mainstream in date palm research. Among the technologies that have been deployed, remote sensing and GIS applications are the most prevalent, accounting for 17.1% and 14.0% of articles respectively. The prominence of these geospatial technologies reflects the spatial nature of date palm agriculture in the Arabian Peninsula, where cultivation is concentrated in discrete oasis well suited to satellite and aerial monitoring. Foundational studies by [74] and [75] established methodological templates for mapping cultivation areas and assessing salinity impacts, frameworks that have been widely adopted by subsequent researchers.

The emergence of AI and machine learning as the fastest-growing technology domain, increasing from zero articles before 2023 to six during 2023–2025, represents a notable shift toward more sophisticated analytical approaches. In date palm systems, AI/ML applications have focused primarily on pest detection, including sensor-based early detection of red palm weevil infestations [76,77]. However, broader adoption will require addressing limitations related to small and geographically localized datasets, cultivar and environmental variability, and potential domain shifts. Future studies should prioritize diverse datasets and independent external validation across cultivars, locations and seasons to improve model generalizability. Greater emphasis on explainable AI, computational efficiency, standardized reporting, and sharing of datasets and code where feasible would further enhance interpretability, reproducibility and practical deployment. These advances will be essential for translating AI/ML from proof-of-concept studies into reliable and scalable decision-support tools for date palm production.

In contrast, the relatively low representation of IoT and sensor technologies (13.4%) warrants careful consideration. The disparity between the promise of IoT and its limited deployment reflects several practical constraints specific to the Arabian Peninsula context. Extreme environmental conditions, including ambient temperatures exceeding 50 °C and high levels of dust, present significant challenges for reliable sensor operation. Additionally, the spatial configuration of date palm cultivation complicates the deployment of point-based monitoring systems and limited cellular connectivity in remote oasis areas constrains data transmission. As [78] noted, connectivity infrastructure represents a fundamental constraint on IoT adoption in the region, suggesting that deployment will require not only technological adaptation but also investments in rural connectivity infrastructure.

The research topic analysis identified water and irrigation management as the most frequently addressed domain (*n* = 76), reflecting the centrality of water scarcity as a constraint on date palm production in the Arabian Peninsula. This finding aligns with the hydrological realities of the region, where annual rainfall rarely exceeds 100 mm and groundwater resources are being depleted at rates far exceeding natural recharge [79]. The convergence of water management research with emerging AI/ML methodologies in the post–2022 period suggests that researchers are increasingly exploring data-driven approaches for optimizing irrigation scheduling, a development that may yield practical tools for improving water use efficiency [80]. Prior work demonstrated that deficit irrigation strategies could reduce water application by up to 30% without compromising date fruit yield or quality, providing evidence that water use efficiency gains are achievable through improved management practices.

The relatively low representation of smart farming and IoT research topics (11.0%), despite their identification as priority areas in national food security strategies, represents a significant disconnect between policy rhetoric and research reality. While GCC nations have articulated ambitious visions for agricultural technology adoption, the research output documented in this analysis suggests that these policy commitments have not yet translated into a commensurate expansion of smart agriculture research for date palm systems. Ref. [81], in their assessment of smart agriculture readiness in the Gulf region, identified connectivity infrastructure as a fundamental constraint on IoT adoption, noting that many agricultural areas lack the telecommunications infrastructure required for real time data transmission. This disconnect underscores the need for stronger mechanisms to align research investments with national strategic priorities.

Keyword analysis and thematic clustering revealed four principal research themes: a geospatial cluster focused on remote sensing and mapping, a water management cluster addressing irrigation and salinity, a pest management cluster targeting red palm weevil and Dubas bug, and an emerging sustainability cluster reflecting broader environmental and food security concerns. The presence of a distinct pest management cluster reflects the severity of the red palm weevil threat and the substantial research investment directed toward developing detection and control methods. Foundational studies by [48,82] established methodological templates for geospatial analysis in date palm systems, demonstrating the utility of remote sensing for mapping cultivation areas and assessing salinity impacts. However, the relatively low representation of disease/pest detection technology as a formal technology category (4.9%) suggests that while pest management is a recognized research topic, the specific application of smart agriculture technologies to pest detection remains limited, representing an opportunity for future research.

The journal analysis revealed a highly dispersed publication landscape, with 164 articles distributed across 117 unique journals and a remarkably low Herfindahl concentration index. This pattern reflects the multidisciplinary nature of date palm smart agriculture research, which spans traditional agronomy, remote sensing, water management and broader sustainability venues. The discrepancy between journal prolificacy and citation impact, with Sustainability being the most productive venue but not among the top five by citation impact, suggests that researchers targeting high citation visibility may benefit from publishing in specialized agricultural technology journals such as Computers and Electronics in Agriculture and Agricultural Water Management. This finding aligns with broader patterns in citation analysis, wherein specialized journals often achieve higher impact factors within their domains than generalist journals [83].

Citation impact analysis revealed significant disparities across technology categories, with precision agriculture articles achieving the highest mean citation count (19.88), followed by GIS/Geospatial (18.61) and IoT/Sensors (15.36). These patterns suggest that articles employing specialized smart agriculture technologies tend to attract greater scholarly attention, reflecting the broader trend in agricultural science wherein technology-focused research often achieves higher visibility [84]. However, the higher citation counts observed for internationally collaborative articles should be interpreted as an association rather than a causal relationship, as citation impact may also reflect publication year, journal visibility, research topic and author networks [85]. This finding highlights the importance of considering collaboration patterns alongside other factors when interpreting research impact, rather than attributing differences in citation counts to collaboration alone.

Several limitations should be acknowledged. First, the restriction to English-language publications may have excluded relevant research published in Arabic or other regional languages, potentially biasing the analysis toward internationally oriented research. Second, reliance on Scopus and Web of Science may have missed research published in regional databases or open access platforms not indexed in these commercial databases. Third, the classification of technology categories and research topics involved subjective judgments, though applied systematically. Fourth, citation impact analysis is inherently time-dependent, a limitation partially addressed through non-parametric methods. These limitations are consistent with those noted in comparable bibliometric studies of agricultural research systems [86,87].

Despite these limitations, the present analysis makes several important contributions. The identification of the persistent technology gap, with field experiments and smart farming/IoT applications being the least represented study design and research topic, respectively, provides a clear roadmap for future research investments. The finding that international collaboration significantly enhances citation impact underscores the value of cross border partnerships in advancing the field [88]. The temporal analysis revealing the rapid emergence of AI/ML research suggests that this domain represents a frontier for future innovation, consistent with global trends documented in agricultural AI research [89]. Collectively, these findings enable researchers, funders and policymakers to track the evolution of the field and assess whether research priorities are aligning with strategic needs.

## 5. Conclusions

This bibliometric study provides a systematic assessment of smart-agriculture research related to date palm (*Phoenix dactylifera* L.) production across the Arabian Peninsula during 2016–2026. The findings indicate that the field is expanding but remains in a transitional stage, with technological development, field validation, and integration into farm-management systems progressing at different rates. Remote sensing/UAV and GIS/geospatial technologies currently represent the most established research domains, particularly for plantation mapping, vegetation monitoring, salinity assessment, and spatial variability analysis. However, their greater research maturity reflects a stronger evidence base rather than widespread readiness for automated farm-scale decision-making. IoT and sensor technologies occupy an intermediate position. Despite their potential for continuous monitoring of soil, irrigation, microclimate, and plant conditions, their adoption remains limited by challenges including heat, dust, salinity, spatial heterogeneity, connectivity, and the need for interoperable systems. AI and machine learning are the fastest-emerging technologies, especially for image-based detection, classification, prediction, and decision support. Nevertheless, their practical maturity remains limited by small or localized datasets, insufficient external validation, seasonal variability, and differences among cultivars and production environments. Future AI/ML research should therefore emphasize larger and more diverse datasets, independent validation, explainability, robustness, and real-world farm testing. A major finding is the integration gap between individual technologies and complete smart-farming systems. Nearly half of the corpus was classified under General Agronomy/Other, while Smart Farming/IoT represented only 11.0%. Similarly, observational and monitoring studies accounted for 50.0% of the corpus, compared with only 6.1% for field experiments. These results suggest that the key challenge is shifting from technological proof-of-concept toward experimentally validated and operational systems. Future research should prioritize multi-season and multi-location field validation, interoperability among sensors, UAV/satellite data, weather information, irrigation systems and analytical platforms, and externally validated AI-based decision support. Water management should remain a major focus given the region’s water constraints, with greater emphasis on testing sensor- and AI-assisted irrigation under real farm conditions. Technology assessment should also consider economic feasibility, maintenance, energy use, connectivity, data governance, and farmer usability. Finally, long-term collaborative field platforms and shared datasets are needed to support standardized benchmarking and independent validation. Collectively, date palm smart-agriculture research is progressing toward integrated digital agriculture, but widespread field-scale validation and operational maturity have not yet been achieved. Future progress should therefore be measured not only by new technologies and publications, but by the successful integration of validated, interoperable, economically feasible, and farmer-relevant solutions into practical date palm management systems.

## Figures and Tables

**Table 1:** Inclusion and exclusion criteria for the bibliometric analysis.

Criterion Type	Description
Inclusion	Published in peer-reviewed journals (original research articles and review articles).
Inclusion	Published between 2016 and 2026 (inclusive).
Inclusion	Written in English.
Inclusion	Focused on date palm (*Phoenix dactylifera* L.) or its cultivation systems as the primary subject.
Inclusion	Study area within the Arabian Peninsula or GCC countries (Saudi Arabia, UAE, Oman, Kuwait, Qatar, Bahrain) or adjacent countries within the broader Arabian Peninsula geography (Iraq, Jordan, Yemen).
Inclusion	Addressed at least one relevant thematic domain: agricultural management, food security, smart agriculture technologies, water/irrigation management, genetics and breeding, pest and disease management, remote sensing, IoT, AI/ML or GIS.
Exclusion	Conference proceedings, book chapters, theses, patents, editorials, letters, and other non-journal publications. Exception: articles first presented at a conference and later published in a peer-reviewed journal.
Exclusion	Articles not written in English.
Exclusion	Studies published before 2016, to maintain focus on contemporary smart agriculture developments.
Exclusion	Articles in which date palm was only incidentally mentioned and not the primary subject of the study.
Exclusion	Studies conducted exclusively outside the defined geographic scope (Arabian Peninsula and adjacent countries).
Exclusion	Duplicate records identified across databases.

**Table 2:** Bibliometric variables extracted for each article in the corpus.

No.	Variable	Description
1	Record-ID	Unique sequential identifier assigned to each article.
2	Source-Database	Database of origin: Scopus or Web of Science.
3	Title	Full article title as indexed in the source database.
4	Authors	Full author list (all contributing authors).
5	Number of Authors	Total count of authors per article.
6	Publication Year	Year of publication (integer, 2016–2026).
7	Journal/Source Title	Title of the journal in which the article was published.
8	Volume and Issue	Volume and issue number of the journal.
9	DOI	Digital Object Identifier-unique persistent identifier.
10	Times Cited	Total citation count at the time of database export.
11	Number of References	Number of references cited by the article.
12	Country of Corresponding Author	Country of affiliation of the corresponding author.
13	Region	Classified as GCC/Arabian Peninsula (non-GCC)/Outside Arabian Peninsula.
14	Number of Countries in Affiliations	Count of distinct countries in author affiliations.
15	International Collaboration	Binary variable (Yes/No); Yes if ≥2 countries appear in author affiliations.
16	Document Type	Article or Review, as classified by the source database.
17	Study Design	Classified according to the framework described in Section 2.2 (String) and Section 2.3.
18	Technology Category	Classified according to the framework described in Section 2.2 and Section 2.3 (multi-label).
19	Research Topic	Classified according to the framework described in Section 2.2 (String) and Section 2.3 (multi-label).
20	Open Access Status	Binary variable (Yes/No) indicating open access availability.
21	Author Keywords	Keywords provided by the article authors.
22	Index Keywords	Keywords assigned by the database indexing system.
23	Abstract	Full text of the article abstract.

**Table 3:** Summary of descriptive statistics for the reviewed corpus (*n* = 164).

Parameter	Value
Total articles included	164
Year range	2016–2026 (partial)
Unique journals	117
Unique countries	21
Open Access articles	126 (76.8%)
Document type: Original article	150 (91.5%)
Document type: Review	9 (5.5%)
Total citations	1911
Mean citations per article (SD)	11.65 (17.77)
Median citations	5
h-index of corpus	23
Most cited article	165 citations [48]
Highly cited articles (≥20 citations)	27 (16.5%)
Uncited articles	29 (17.7%)
International collaboration rate	62 articles (37.8%)
GCC contribution	93 articles (56.7%)
Saudi Arabia contribution	54 articles (32.9%)
Linear trend slope (β)	1.25 articles/year (R^2^ = 0.358, *p* = 0.068)
Mann–Kendall trend test	τ = 0.489, z = 1.878, *p* = 0.060

**Table 4:** Top 10 most cited articles in the reviewed corpus.

Rank	Article	Year	Country	Citations
1	[53]	2009	UAE	165
2	[54]	2022	UAE	147
3	[55]	2022	UAE	138
4	[56]	2022	UK	136
5	[57]	2008	UAE	105
6	[58]	2018	UAE	99
7	[59]	2006	UAE	94
8	[60]	2019	UAE	90
9	[61]	2021	KSA	85
10	[62]	2020	UAE	81

## Data Availability

The original contributions presented in this study are included in the article. Further inquiries can be directed to the corresponding author.

## References

[1] Gorka R., Kumar R. (2026). Geographical and Climatic Characteristics of the Arabian Peninsula: Evolution of Landforms and Climate Regimes. Climate Change in the Arabian Peninsula: Challenges and Pathways to Solutions.

[2] Ghazanfar S.A. (2024). Biogeography and conservation in the Arabian Peninsula: A present perspective. Plants.

[3] Kumari N., Hembrom S., Pandey S. (2026). Biodiversity and Ecosystem Dynamics of the Arabian Peninsula: Climate-Driven Changes, Species Vulnerability and Conservation Strategies. Climate Change in the Arabian Peninsula: Challenges and Pathways to Solutions.

[4] Sayed O.H., Masrahi Y.S. (2023). Climatology and phytogeography of Saudi Arabia. A review. Arid. Land Res. Manag..

[5] Dawoud M.A. (2025). Groundwater management and governance in MENA region. Groundwater in Developing Countries: Case Studies from MENA, Asia and West Africa.

[6] Safeer S., Rahmatullah Q., Saeed K., Farhan A. (2017). Ethnobotanical study on useful indigenous plants in Mahasheer National Park, AJK. J. Coast Life Med..

[7] Al-Rashidi A.F., Alnagar D.K., Eledum H. (2025). Forecasting water consumption for sustainable development in Saudi Arabia: A copula-based approach. Water.

[8] Hassen T.B., El Bilali H. (2019). Food security in the Gulf Cooperation Council countries: Challenges and prospects. J. Food Secur..

[9] Elmi A.A. (2017). Food Security in the Arab Gulf Cooperation Council States. Sustainable Agriculture Reviews.

[10] Al-Saidi M., Saliba S. (2019). Water, energy and food supply security in the Gulf Cooperation Council (GCC) countries—A risk perspective. Water.

[11] Jaradat A.A. (2015). Biodiversity, genetic diversity, and genetic resources of date palm. Date Palm Genetic Resources and Utilization: Volume 1: Africa and the Americas.

[12] Ali S., Ahmad F., Mohammad Z.H., Ibrahim S.A., Javed F. (2025). Global History of Dates. Date Fruits and By-Products Processing.

[13] Gros-Balthazard M., Battesti V., Flowers J.M., Ferrand S., Breil M., Ivorra S., Bourgeois Y. (2023). What lies behind a fruit crop variety name? A case study of the barnī date palm from al-‘Ulā oasis, Saudi Arabia. Plants People Planet.

[14] Al-Habsi N. (2025). Date palm (*Phoenix dactylifera* L.) fruit: Strategic crop for food security, nutritional benefits, postharvest quality, and valorization into emerging functional products. Sustainability.

[15] Al-Moftah A.M.S., Alnajideen M., Alafifi F., Czyzewski P., Shi H., Alherbawi M., Valera-Medina A. (2025). Economic feasibility of using municipal solid waste and date palm waste for clean energy production in Qatar. Energies.

[16] Al-Mssallem M.Q., Al-Khayri J.M., Alghamdi B.A., Alotaibi N.M., Alotaibi M.O., Al-Qthanin R.N., Al-Shalan H.Z. (2024). Role of date palm to food and nutritional security in Saudi Arabia. Food and Nutrition Security in the Kingdom of Saudi Arabia, Vol. 2: Macroeconomic Policy and Its Implication on Food and Nutrition Security.

[17] Keskin B., Uzundumlu A.S., Karabacak T. (2025). Production estimates of leading countries in date production for the period 2023–2028. Appl. Fruit. Sci..

[18] Emam A. (2025). Climate Change Effects on Dates Productivity in Saudi Arabia: Implications for Food Security. Sustainability.

[19] Almutawa A.A. (2022). Date production in the Al-Hassa region, Saudi Arabia in the face of climate change. J. Water Clim. Change.

[20] Świąder K., Białek K., Hosoglu I. (2020). Varieties of date palm fruits (*Phoenix dactylifera* L.), their characteristics and cultivation^®^. Postępy Tech. Przetwórstwa Spoż..

[21] Nazri M.K.N.Z., Yusoff A.M., Nazri N.F.Z., Khalaf N.A.R., Rahman A.A. (2016). The descriptions of date palms and an ethnomedicinal importance of dates mentioned in the quran. Mediterr. J. Soc. Sci..

[22] Hamriri K., Atmani M., Abidar A., Aziz L., Fagroud M., Bouamri R. (2024). Sustainable oases agriculture: A journey through Morocco’s date palm production system. J. Water Land Dev..

[23] Khanum P., Khan A.A., Khan I.A., Ghaffar A., Khan Z. (2025). Recent advances in date palm (*Phoenix dactylifera* L.) Biotechnology and breeding. Breeding and Biotechnology of Leaf, Fruit, and Seed Fiber Crops.

[24] Rahman H., Vikram P., Hammami Z., Singh R.K. (2022). Recent advances in date palm genomics: A comprehensive review. Front. Genet..

[25] Ijaz S., Babar M. (2020). Ornamental palms: Molecular taxonomy, ecology and distribution. Etiology and Integrated Management of Economically Important Fungal Diseases of Ornamental Palms.

[26] Safeer S., Gallo P., Pulvento C. (2025). Agri-farming with computer vision, IoT and blockchain towards climate smart cultivation. Internet Things.

[27] Safeer S., Lemieux V., Lu C., Pulvento C. (2026). BIoT-DApp: A Prototype for Real Time Traceability in Agricultural Supply Chains. Emerg. Sci. J..

[28] Robin-Soriano A., Vincent B., Maurice K., Battesti V., Boukcim H., Ducousso M., Gros-Balthazard M. (2025). Digging deeper into the impacts of different soil water systems on the date palm root architecture and associated fungal communities. Symbiosis.

[29] Obón C., Rivera D., Amorós A., Díaz G., Alcaraz F., Johnson D.V. (2023). Botany and physiology of date palm. Date Palm..

[30] Awad M.A., Al-Qurashi A.D., Mohamed S.A. (2011). Biochemical changes in fruit of an early and a late date palm cultivar during development and ripening. Int. J. Fruit. Sci..

[31] Haider M.S., Khan I.A., Jaskani M.J., Naqvi S.A., Mateen S.A.J.I.D., Shahzad U., Abbas H.A.I.D.E.R. (2018). Pomological and biochemical profiling of date fruits (*Phoenix dactylifera* L.) during different fruit maturation phases. Pak. J. Bot..

[32] Chao C.T., Krueger R.R. (2007). The date palm (*Phoenix dactylifera* L.): Overview of biology, uses, and cultivation. HortScience.

[33] Alotaibi K.D., Alharbi H.A., Yaish M.W., Ahmed I., Alharbi S.A., Alotaibi F., Kuzyakov Y. (2023). Date palm cultivation: A review of soil and environmental conditions and future challenges. Land Degrad. Dev..

[34] Safronov O., Kreuzwieser J., Haberer G., Alyousif M.S., Schulze W., Al-Harbi N., Kangasjärvi J. (2017). Detecting early signs of heat and drought stress in *Phoenix dactylifera* (date palm). PLoS ONE.

[35] Safeer S., Mastro G.D., Pulvento C. (2024). IoT based climate smart agriculture succeeded by blockchain database—A bibliometric analysis. Front. Sustain. Food Syst..

[36] Pranckutė R. (2021). Web of Science (WoS) and Scopus: The titans of bibliographic information in today’s academic world. Publications.

[37] Aria M., Cuccurullo C. (2017). bibliometrix: An R-tool for comprehensive science mapping analysis. J. Informetr..

[38] Niaz J., Scaringi G., Cagnazzo C., Parise M., Lollino P. (2026). Research Trends in Thermal Surveys and Thermomechanical Modeling of Landslides. Appl. Sci..

[39] Van Bommel H.M., Hubers F., Maas K.E.H. (2024). Prominent themes and blind spots in diversity and inclusion literature: A bibliometric analysis. J. Bus. Ethics.

[40] Veiga-del-Baño J.M., Pérez-Lucas G., Andreo-Martínez P., Navarro S. (2025). Bibliometric analysis on graphitic carbon nitride (g-C_3_N_4_) as photocatalyst for the remediation of water polluted with contaminants of emerging concern. Catalysts.

[41] Dervis H. (2019). Bibliometric analysis using bibliometrix an R package. J. Sci. Res..

[42] Marshall G., Jonker L. (2011). An introduction to inferential statistics: A review and practical guide. Radiography.

[43] Bandyopadhyay D., Richard E. (2012). Plant: Spatial Data Analysis in Ecology and Agriculture Using R. J. Agric. Biol. Environ. Stat..

[44] Da Silva R.M., Santos C.A., Moreira M., Corte-Real J., Silva V.C., Medeiros I.C. (2015). Rainfall and river flow trends using Mann–Kendall and Sen’s slope estimator statistical tests in the Cobres River basin. Nat. Hazards.

[45] Romero-Organvidez D., Horcas J.M., Galindo J.A., Benavides D. (2024). Data visualization guidance using a software product line approach. J. Syst. Softw..

[46] Borgohain D.J., Verma M.K., Nazim M., Sarkar M. (2021). Application of Bradford’s law of scattering and Leimkuhler model to information science literature. COLLNET J. Scientometr. Inf. Manag..

[47] Brezina I., Pekár J., Čičková Z., Reiff M. (2016). Herfindahl–Hirschman index level of concentration values modification and analysis of their change. Cent. Eur. J. Oper. Res..

[48] Dahy B., Issa S., Saleous N. (2023). Geo-spatial modelling of carbon stock assessment of date palm at different age stages: An integrated approach of fieldwork, remote sensing and GIS. Ecol. Model..

[49] Yeo-Teh N.S.L., Tang B.L. (2022). Sustained rise in retractions in the life sciences literature during the pandemic years 2020 and 2021. Publications.

[50] Yusuf N., Kooli C., Khoj H., Bajnaid N.O. (2025). Strategizing sustainable food security in Saudi Arabia: A policy and scenario approach to agricultural resilience. Outlook Agric..

[51] Alhammad Z., Awaideh M. (2023). Exploring the role of dates value chains in enhancing food security in Jordan. Res. J. Agric. Biol. Sci..

[52] Pandey N., Tripathi P., Pandey N., Nakum H., Vala Y.S. (2024). The sustainable improvement and precision agriculture in the date palm cultivation—A Review. J. Agric. Ecol..

[53] Habib H.M., Ibrahim W.H. (2009). Nutritional quality evaluation of eighteen date pit varieties. Int. J. Food Sci. Nutr..

[54] Airouyuwa J.O., Mostafa H., Riaz A., Maqsood S. (2022). Utilization of natural deep eutectic solvents and ultrasound-assisted extraction as green extraction technique for the recovery of bioactive compounds from date palm (*Phoenix dactylifera* L.) seeds: An investigation into optimization of process parameters. Ultrason. Sonochem..

[55] Raza M., Abu-Jdayil B., Banat F., Al-Marzouqi A.H. (2022). Isolation and characterization of cellulose nanocrystals from date palm waste. ACS Omega.

[56] Jintasuttisak T., Edirisinghe E., Elbattay A. (2022). Deep neural network based date palm tree detection in drone imagery. Comput. Electron. Agric..

[57] Alhammadi M.S., Glenn E.P. (2008). Detecting date palm trees health and vegetation greenness change on the eastern coast of the United Arab Emirates using SAVI. Int. J. Remote Sens..

[58] Aldababseh A., Temimi M., Maghelal P., Branch O., Wulfmeyer V. (2018). Multi-criteria evaluation of irrigated agriculture suitability to achieve food security in an arid environment. Sustainability.

[59] Ismail B., Haffar I., Baalbaki R., Mechref Y., Henry J. (2006). Physico-chemical characteristics and total quality of five date varieties grown in the United Arab Emirates. Int. J. Food Sci. Technol..

[60] Makkawi Y., El Sayed Y., Salih M., Nancarrow P., Banks S., Bridgwater T. (2019). Fast pyrolysis of date palm (*Phoenix dactylifera*) waste in a bubbling fluidized bed reactor. Renew. Energy.

[61] Almusallam I.A., Ahmed I.A.M., Babiker E.E., Al Juhaimi F.Y., Fadimu G.J., Osman M.A., Alqah H.A. (2021). Optimization of ultrasound-assisted extraction of bioactive properties from date palm (*Phoenix dactylifera* L.) spikelets using response surface methodology. LWT.

[62] Martis R., Al-Othman A., Tawalbeh M., Alkasrawi M. (2020). Energy and economic analysis of date palm biomass feedstock for biofuel production in UAE: Pyrolysis, gasification and fermentation. Energies.

[63] Tennant J.P., Waldner F., Jacques D.C., Masuzzo P., Collister L.B., Hartgerink C.H. (2016). The academic, economic and societal impacts of Open Access: An evidence-based review. F1000Research.

[64] Verma S., Sharma H. (2024). Quantity over quality of publications: Are we using the right metrics to judge author’s productivity and impact in biomedical research?. J. Postgrad. Med..

[65] Iqbal A. (2025). Comprehensive AI framework for automated classification, detection, segmentation, and severity estimation of date palm diseases using vision-language models and generative AI. Front. Plant Sci..

[66] Alhaider I., Ahmed M.K., Gupta B.M. (2014). Global research output on date palm (*Pheonix dactylifera*): A 12 years scientometric perspective. Scientometrics.

[67] El Chami D., Daccache A., El Moujabber M. (2020). How can sustainable agriculture increase climate resilience? A systematic review. Sustainability.

[68] Naeini M.S., Nazari B., Liaghat A. (2025). Developing a hierarchical analytical process to determine the best irrigation system for date palm trees in the NENA region: The case of Iran. Appl. Water Sci..

[69] Wu Y., Meng S., Liu C., Gao W., Liang X.Z. (2023). A bibliometric analysis of research for climate impact on agriculture. Front. Sustain. Food Syst..

[70] El-Habba M.S., Al-Mulhim F. (2013). The competitiveness of the Saudi Arabian date palm: An analytical study. Afr. J. Agric. Res..

[71] Gajdić D., Kotzab H., Petljak K. (2023). Collaboration, trust and performance in agri-food supply chains: A bibliometric analysis. Br. Food J..

[72] Klerkx L., Jakku E., Labarthe P. (2019). A review of social science on digital agriculture, smart farming and agriculture 4.0: New contributions and a future research agenda. NJAS Wagening. J. Life Sci..

[73] El-Hendawy S., Alsamin B., Mohammed N., Al-Suhaibani N., Refay Y., Alotaibi M., Mattar M.A. (2022). Combining planting patterns with mulching bolsters the soil water content, growth, yield, and water use efficiency of spring wheat under limited water supply in arid regions. Agronomy.

[74] Zolfaghary P., Zakerinia M., Kazemi H. (2021). A model for the use of urban treated wastewater in agriculture using multiple criteria decision making (MCDM) and geographic information system (GIS). Agric. Water Manag..

[75] Allbed A., Kumar L., Sinha P. (2018). Soil salinity and vegetation cover change detection from multi-temporal remotely sensed imagery in Al Hassa Oasis in Saudi Arabia. Geocarto Int..

[76] Saleem M.H., Potgieter J., Arif K.M. (2021). Automation in agriculture by machine and deep learning techniques: A review of recent developments. Precis. Agric..

[77] Domingues T., Brandão T., Ferreira J.C. (2022). Machine learning for detection and prediction of crop diseases and pests: A comprehensive survey. Agriculture.

[78] Rafi M.S.M., Behjati M., Rafsanjani A.S. (2025). Reliable and cost-efficient IoT connectivity for smart agriculture: A comparative study of LPWAN, 5G, and hybrid connectivity models. International Conference on Smart Computing and Informatics.

[79] Taylor R.G., Todd M.C., Kongola L., Maurice L., Nahozya E., Sanga H., MacDonald A.M. (2013). Evidence of the dependence of groundwater resources on extreme rainfall in East Africa. Nat. Clim. Change.

[80] Al-Muaini A., Green S., Dakheel A., Abdullah A.H., Abou Dahr W.A., Dixon S., Kemp P., Clothier B. (2019). Irrigation management with saline groundwater of a date palm cultivar in the hyper-arid United Arab Emirates. Agric. Water Manag..

[81] Al-Ammary J.H., Ghanem M.E. (2024). Information and communication technology in agriculture: Awareness, readiness and adoption in the Kingdom of Bahrain. Arab. Gulf J. Sci. Res..

[82] Benslama A., Khanchoul K., Benbrahim F., Boubehziz S., Chikhi F., Navarro-Pedreño J. (2020). Monitoring the variations of soil salinity in a palm grove in Southern Algeria. Sustainability.

[83] Wezel A., Soldat V. (2009). A quantitative and qualitative historical analysis of the scientific discipline of agroecology. Int. J. Agric. Sustain..

[84] Kamilaris A., Prenafeta-Boldú F.X. (2018). A review of the use of convolutional neural networks in agriculture. J. Agric. Sci..

[85] Guan J., Yan Y., Zhang J.J. (2017). The impact of collaboration and knowledge networks on citations. J. Informetr..

[86] Yang G., Li J., Liu Z., Zhang Y., Xu X., Zhang H., Xu Y. (2022). Research trends in crop–livestock systems: A bibliometric review. Int. J. Environ. Res. Public Health.

[87] Bertoglio R., Corbo C., Renga F.M., Matteucci M. (2021). The digital agricultural revolution: A bibliometric analysis literature review. IEEE Access.

[88] Yadigarova L. (2025). Bibliometric analysis of the impact of international research collaborations: Insights from social sciences. Int. Sci. J. Univ. Leadersh..

[89] Ruiz-Real J.L., Uribe-Toril J., Torres Arriaza J.A., de Pablo Valenciano J. (2020). A look at the past, present and future research trends of artificial intelligence in agriculture. Agronomy.

